# Supplementary material for: A genome-wide association study identifies EYA2 as a contributing gene for diabetic retinopathy in type 2 diabetes
Source: Commun Med (Lond). 2026 Feb 25;6:181. doi: 10.1038/s43856-026-01465-1 (PMC13046729; doi:10.1038/s43856-026-01465-1)
Supplement: Supplementary file 2 — Supplementary File [file 43856_2026_1465_MOESM2_ESM.pdf]

A genome-wide association study identifies *EYA2* as a contributing gene for diabetic retinopathy in type 2 diabetes

Supplementary Information

**Supplementary Table 1: Clinical Characteristics of GWAS Participants Derived from the UK Biobank**			
Covariates	***Cases***	***Controls***	***P***
Sex(male:female)	1242:582(68.09%:31.91%)	9823:5341(64.78%:35.22%)	5.02e-3 < 0.01
Age(years)	61.7(6.04)	60.7(6.46)	1.13e-10 < 0.01
BMI(kg/m^2)	32.4(5.60)	31.9(5.74)	6.14e-04 < 0.01
DoD(duration)	11.2(10.12)	6.7(8.15)	2.20e-16 < 0.01

A chi-square test (two-sided) assessed the differences in gender distribution between cases and controls, while an independent t-test (two-sided) evaluated other covariates.
Continuous covariates were shown as 'mean(standard deviation)'.

**Supplementary Table 2. Replication cohorts' information**						
**Cohorts**	**Studied Trait**	**Diabetic retinopathy ascertainment**	**Definition of Cases and Controls**	**Discovery Sample Size (N)**		
				**Cases**	**Controls**	**Total**
GoDARTS (Rajendrakumar 2022)	DR	Any DR was defined as the first reported diabetic retinopathy of R0 or above in the Scottish grading scheme. For GWAS analysis, selected 'accelerated DR' cases developing the condition within 10 years. Controls were the individuals without DR and had more than 10 years of follow-up. In the GWAS study, T1D: 107, T2D: 4,906	**Case**: samples with any DR in diabetes mellitus<br>**Control**: samples without DR in diabetes mellitus	3,222	1,791	5,013
FinnGen (Kurki et al. 2023)	DR	In FinnGen Freeze 5, diabetic retinopathy is defined by the PheWeb endpoint H7_RETINOPATHYDIAB, which encompasses all ICD-10 H36 subcodes (H36.0–H36.3) under the "VII Diseases of the eye and adnexa (H7_)" category—specifically background retinopathy (H7_RETINOPATHYDIAB_BKG), diabetic maculopathy (H7_MACULOPATHYDIAB), severe background DR (H7_RETINOPATHYDIAB_BKG_SEVERE), proliferative DR (H7_RETINOPATHYDIAB_PROLIF), advanced DR (H7_RETINOPATHYDIAB_ADVANC), DR consequences (H7_RETINOPATHYDIAB_CONSEQ), and unspecified DR (H7_RETINOPATHYDIAB_NAS). Cases are any individuals with one or more of these registry-based diagnoses in the Finnish hospital discharge, outpatient, or cause-of-death registers; controls are participants with no record of H7_RETINOPATHYDIAB.	**Case**: samples with DR from general population.<br>**Control**: samples without DR from general population	3,646	203,018	206,664
African American ancestry (Verma et al. 2024)	DR	Diabetic retinopathy was defined using the PheCode 250.7 ("Diabetic retinopathy") within the Endocrine/metabolic category. Cases were those with ≥2 instances of PheCode 250.7 in their electronic health records, and controls were individuals with no record of any 250.x diabetes-related PheCode. Individuals with only one PheCode 250.7 record were excluded.	**Case**: samples with DR from general population.<br>**Control**: samples without diabetes-related phenotypes from general population	11,781	103,431	115,212

European ancestry (Verma et al. 2024)	DR	Diabetic retinopathy was defined using the PheCode 250.7 ("Diabetic retinopathy") within the Endocrine/metabolic category. Cases were those with ≥2 instances of PheCode 250.7 in their electronic health records, and controls were individuals with no record of any 250.x diabetes-related PheCode. Individuals with only one PheCode 250.7 record were excluded.	**Case**: samples with DR from general population.<br>**Control**: samples without diabetes-related phenotypes from general population	29,668	402,541	432,209

**Supplementary Table 3: Parameters setting in FUMA platform**					
FUMA	v1.5.2	***genetype***	protein_coding	***eqtlMapP***	1
MAGMA	v1.08	***leadP***	5.00E-08	***ciMapBuiltin***	Selece All
GWAScatalog	e0_r2022-11-29	***gwasP***	0.05	***ciMapFileN***	0
ANNOVAR	2017/7/17	***r2***	0.6	***ciMapFiles***	NA
becol	beta	***r2_2***	0.1	***ciMapFDR***	1.00E-06
secol	se	***refpanel***	UKB/release2b	***ciMapPromWindow***	250-500
leadSNPsfile	NA	***pop***	WBrits_10k	***ciMapRoadmap***	default
addleadSNPs	1	***MAF***	0	***ciMapEnhFilt***	0
regionsfile	NA	***refSNPs***	1	***ciMapPromFilt***	0
GRCh38	0	***mergeDist***	250	***ciMapCADDth***	0
N	NA	***magma***	1	***ciMapRDBth***	NA
Ncol	N	***magma_window***	42.5	***ciMapChr15***	NA
exMHC	1	***magma_exp***	GTEx/v8/gtex_v8_ts_avg_log2TPM, GTEx/v8/gtex_v8_ts_general_avg_log2TPM	***ciMapChr15Max***	NA
MHCopt	annot	***eqtlMap***	1	***ciMapChr15Meth***	NA
extMHC	NA	***eqtlMaptss***	Select All	***ciMapAnnoDs***	NA
ensembl	v102	***eqtlMapSig***	1	***ciMapAnnoMeth***	NA
*Other parameters are set to default					

Supplementary Table 4. GWAS ID used in Mendelian Randomization from the IEU OpenGWAS database					
	**General diabetic indicators**		**Related ocular diseases**		
	**Type 2 diabetes**	**Proinsulin**	**Cataract**	**Glaucoma**	**Eyelid disorders**
IEU OpenGWAS GWAS_ID	ebi-a-GCST006867', 'ebi-a-GCST90018926', 'ebi-a-GCST90038634', 'ebi-a-GCST90029024', 'ukb-b-13806', 'ebi-a-GCST010118', 'ebi-a-GCST90013892', 'ebi-a-GCST90013942', 'ukb-a-75', 'ebi-a-GCST007515', 'ebi-a-GCST007517', 'ebi-a-GCST007516', 'ebi-a-GCST007518', 'ebi-a-GCST90018706', 'ieu-a-24', 'ieu-a-1090', 'ieu-a-23', 'ieu-a-25', 'ebi-a-GCST005413', 'ebi-a-GCST005047', 'ieu-a-26', 'ieu-a-976', 'ebi-a-GCST90093110', 'ebi-a-GCST90006934', 'ebi-a-GCST005898', 'ebi-a-GCST008048', 'ebi-a-GCST90026417', 'ebi-a-GCST90026416', 'ebi-a-GCST90026415', 'ebi-a-GCST90026413', 'ebi-a-GCST90026414', 'ebi-a-GCST90026412', 'finn-b-E4_DM2', 'finn-b-E4_DM2COMA', 'finn-b-E4_DM2KETO', 'finn-b-E4_DM2NEU', 'finn-b-E4_DM2OPTH', 'finn-b-E4_DM2NASCOMP', 'finn-b-E4_DM2PERIPH', 'finn-b-E4_DM2REN', 'finn-b-E4_DM2NOCOMP', 'finn-b-T2D', 'finn-b-T2D_INCLAVO', 'finn-b-E4_DM2_STRICT', 'finn-b-T2D_WIDE'	ebi-a-GCST001212'	ukb-b-8329', 'ukb-a-426'	ukb-b-17324', 'ukb-a-424'	ebi-a-GCST90038640', 'ukb-b-9565', 'ukb-b-9058', 'ukb-d-H7_EYELIDDIS', 'ukb-d-H02'

Supplementary Table 5. Replication of top DR-associated SNPs reported in five independent cohorts within our UKB DR GWAS.

Discovery cohorts				Replication cohort (UK Biobank)
**CHR**	**SNP**	**POS**	***P***	***P***
GoDARTS				
19	rs111817537	10707753	3.16E-08	0.730
FinnGen				
6	rs9275618	32684387	2.6E-151	0.593
11	rs689	2182224	1.4E-23	0.216
African American ancestry				
10	rs7903146	114758349	7.31E-72	0.039
11	rs2237897	2858546	7.85E-14	0.037
European ancestry				
10	rs34872471	114754071	3.46E-261	0.049
16	rs1421085	53800954	8.25E-74	0.933
Chinese cohorts				
13	rs9565164	76039376	1.30E-07	0.521
2	rs1399634	170244607	2.00E-06	0.050
2	rs2380261	235641180	2.10E-06	0.428

P values reported for the discovery cohorts (GoDARTS, FinnGen, African American ancestry, European ancestry, and Chinese cohorts) were extracted from the corresponding published GWAS summary statistics. *P* values for the UKB replication column were obtained from our UKB GWAS performed using a generalized linear mixed model. Two-sided *p* values were obtained from the 1 d.f. score test of the SNP effect.

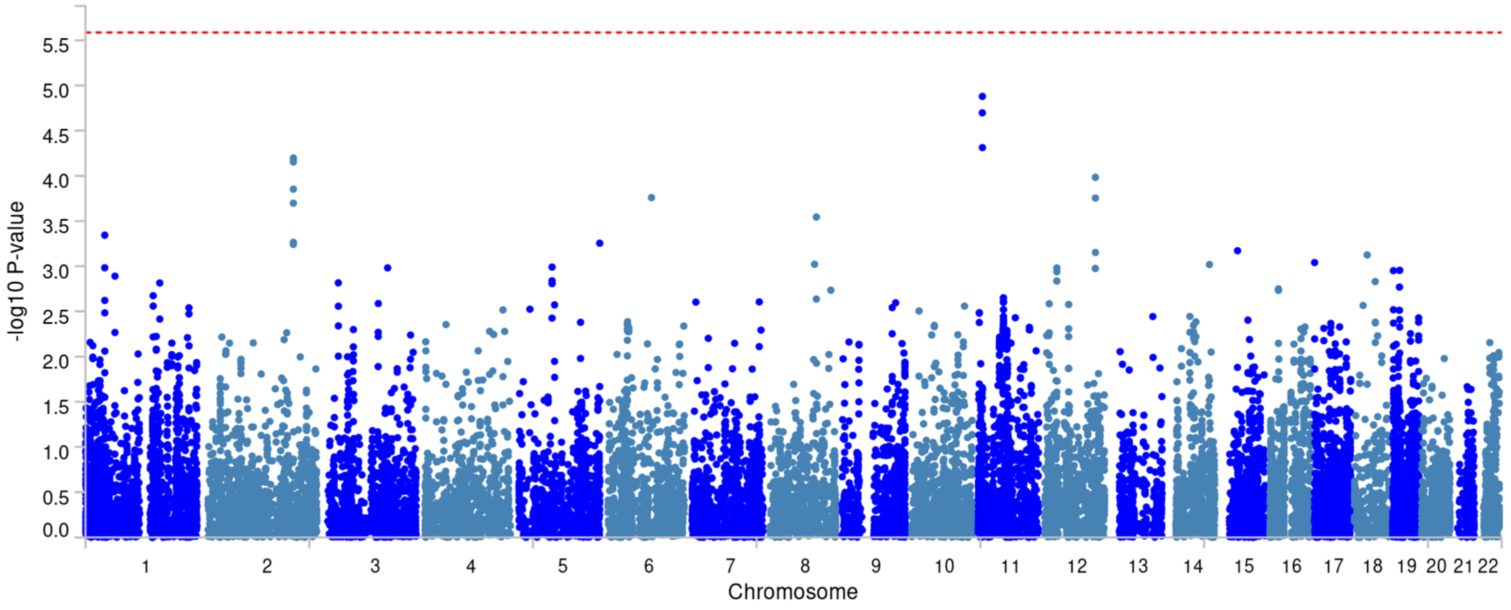

Supplementary Figure 1. The Manhattan plot of the gene-based test based on the GWAS summary statistics.

Genome-wide significance was defined and established after Bonferroni correction (p = 0.05/19295 = 2.59 x10-6), as illustrated by the red dashed line in the plot. It appears that none of the genes reached genome-wide significance.

Supplementary Table 6: The top 30 gene sets from MAGMA Gene-Set Analysis results

Gene Set	*N genes*	*Beta*	*Beta STD*	*SE*	*P*	P_{bon}
GOBP_REGULATION_OF_PRO_B_CELL_DIFFERENTIATION	8	1.438	0.029	0.312	1.98E-06	0.034
BIOCARTA_PS1_PATHWAY	14	0.948	0.026	0.245	5.43E-05	0.924
GOBP_NEGATIVE_REGULATION_OF_CELL_JUNCTION_ASSEMBLY	30	0.578	0.023	0.152	7.54E-05	1
GOBP_ARTERIAL_ENDOTHELIAL_CELL_DIFFERENTIATION	6	1.224	0.022	0.327	9.03E-05	1
GOBP_REGULATION_OF_LYMPHOID_PROGENITOR_CELL_DIFFERENTIATION	12	0.948	0.024	0.254	9.39E-05	1
GOBP_GROWTH_INVOLVED_IN_HEART_MORPHOGENESIS	4	1.477	0.021	0.415	1.86E-04	1
GOBP_POSITIVE_REGULATION_OF_KERATINOCYTE_DIFFERENTIATION	18	0.788	0.024	0.228	2.71E-04	1
GOBP_ATRIOVENTRICULAR_NODE_DEVELOPMENT	8	0.956	0.019	0.278	2.89E-04	1
GOBP_PRO_B_CELL_DIFFERENTIATION	12	0.921	0.023	0.277	4.41E-04	1
GOBP_HISTONE_H2A_K63_LINKED_UBIQUITINATION	6	1.030	0.018	0.311	4.58E-04	1
GOBP_REGULATION_OF_SOMITOGENESIS	6	1.109	0.020	0.335	4.62E-04	1
GOBP_REGULATION_OF_ARTERY_MORPHOGENESIS	4	1.120	0.016	0.338	4.66E-04	1
GOBP_ATRIOVENTRICULAR_VALVE_FORMATION	8	0.967	0.020	0.292	4.74E-04	1
GOBP_POSITIVE_REGULATION_OF_APOPTOTIC_PROCESS_INVOLVED_IN_DEVELOPMENT	5	1.272	0.020	0.387	5.07E-04	1
GOBP_POSITIVE_REGULATION_OF_APOPTOTIC_PROCESS_INVOLVED_IN_MORPHOGENESIS	5	1.272	0.020	0.387	5.07E-04	1
GOBP_ENDOCARDIUM_MORPHOGENESIS	6	0.971	0.017	0.297	5.32E-04	1
BIOCARTA_HES_PATHWAY	12	0.786	0.020	0.242	5.77E-04	1
REACTOME_DEFECTIVE_LFNG_CAUSES_SCDO3	5	0.970	0.016	0.305	7.25E-04	1
GOMF_PYRIMIDINE_NUCLEOSIDE_TRANSMEMBRANE_TRANSPORTER_ACTIVITY	7	0.827	0.016	0.261	7.56E-04	1
GOBP_REGULATION_OF_PROTEIN_LOCALIZATION_TO_CELL_SURFACE	38	0.429	0.019	0.136	7.69E-04	1
GOBP_NEGATIVE_REGULATION_OF_PHOTORECEPTOR_CELL_DIFFERENTIATION	6	1.318	0.023	0.419	8.36E-04	1
GOBP_APOPTOTIC_PROCESS_INVOLVED_IN_MORPHOGENESIS	26	0.512	0.019	0.163	8.60E-04	1
GOBP_NEGATIVE_REGULATION_OF_CELL_PROLIFERATION_INVOLVED_IN_HEART_MORPHOGENESIS	4	1.288	0.019	0.412	8.86E-04	1

GOBP_NEGATIVE_REGULATION_OF_IMMUNOGLOBULIN_PRODUCTION	11	0.650	0.016	0.209	9.52E-04	1
GOMF_INOSITOL_1_4_5_TRISPHOSPHATE_BINDING	11	0.854	0.020	0.275	9.66E-04	1
GOMF_CARNITINE_TRANSMEMBRANE_TRANSPORTER_ACTIVITY	5	1.228	0.020	0.397	9.93E-04	1
GOBP_MESENCHYMAL_CELL_DEVELOPMENT	6	1.017	0.018	0.336	1.24E-03	1
GOBP_HEPARAN_SULFATE_PROTEOGLYCAN_BIOSYNTHETIC_PROCESS_POLYSACCHARIDE_CHAIN_BIOSYNTHETIC_PROCESS	8	0.963	0.020	0.319	1.25E-03	1
GOBP_NEGATIVE_REGULATION_OF_SYNAPSE_ASSEMBLY	8	0.874	0.018	0.291	1.34E-03	1
REACTOME_PRE_NOTCH_PROCESSING_IN_THE_ENDOPLASMIC_RETICULUM	6	0.899	0.016	0.300	1.38E-03	1

Top 30 gene set analysis results by MAGMA. Gene-set enrichment was tested using MAGMA competitive gene-set analysis, which fits a gene-level regression model of gene association statistics on gene-set membership. P values correspond to a one-sided test of the gene-set effect.

Supplementary Table 7: Statistical Details of Genes from GOBP_REGULATION_OF_PRO_B_CELL_DIFFERENTIATION											
GENE_ID	*GENE*	*CHR*	*START*	*STOP*	*NSNPS*	*NPARAM*	*N*	*ZSTAT*	*P*	*ZFITTED_BASE*	*ZRESID_BASE*
ENSG00000197921	*HES5*	1	2417684	2504184	234	26	16988	-1.40	0.92	0	-1.40
ENSG00000115904	*SOS1*	2	39166037	39393986	514	21	16988	2.32	0.01	0	2.32
ENSG00000114315	*SOS1*	3	193811434	193899021	343	46	16988	0.90	0.18	0	0.90
ENSG00000217128	*FNIP1*	5	130934907	131175210	394	18	16988	1.11	0.13	0	1.11
ENSG00000148400	*NOTCH1*	9	139346396	139482814	426	60	16988	0.68	0.24	0	0.68
ENSG00000100485	*SOS2*	14	50541347	50740776	682	29	16988	2.24	0.01	0	2.24
ENSG00000167005	*NUDT21*	16	56420545	56528611	295	23	16988	-0.50	0.68	0	-0.50
ENSG00000154803	*FLCN*	17	17073026	17183002	344	30	16988	-0.30	0.61	0	-0.30

Gene-level P values and ZSTAT were obtained from MAGMA gene analysis using the default SNP-wise mean model for summary statistics; ZSTAT is derived from P via $Z = \Phi^{-1}(1 - P)$. ZFITTED_BASE and ZRESID_BASE are fitted and residual Z values from the base model (intercept + conditioned covariates). The gene set was significant in MAGMA competitive gene-set analysis (one-sided test) after Bonferroni correction across all tested gene sets; per-gene P values shown here are unadjusted and provided for descriptive purposes.

GENE_ID: Ensembl gene identifier; CHR: chromosome; START: start position of the gene; STOP: end position of the gene; NSNPS: number of SNPs mapped to the gene region; NPARAM: number of parameters included in the association model; ZSTAT: Z-statistic; ZFITTED_BASE: fitted baseline Z-score from the MAGMA gene analysis model; ZRESID_BASE: residual Z-score (after fitting the baseline model) from the MAGMA analysis.

Supplementary Table 8: tissue expression analysis results for 30 general and 54 specific tissue types						
**30 general tissue types**						
**VARIABLE**	**TYPE**	**NGENES**	**BETA**	**BETA_STD**	**SE**	**P**
Spleen	COVAR	17379	0.02225	0.044121	0.0076414	0.0017997
Adrenal_Gland	COVAR	17379	0.01481	0.028609	0.010071	0.07072
Small_Intestine	COVAR	17379	0.014296	0.026312	0.010379	0.084194
Cervix_Uteri	COVAR	17379	0.012163	0.023914	0.013674	0.18689
Salivary_Gland	COVAR	17379	0.006011	0.011195	0.010372	0.28112
Breast	COVAR	17379	0.007699	0.014817	0.014992	0.30379
Blood_Vessel	COVAR	17379	0.0048655	0.0098789	0.011385	0.33456
Blood	COVAR	17379	0.0025019	0.0044945	0.0060413	0.33939
Liver	COVAR	17379	0.0024871	0.0044806	0.006591	0.35296
Vagina	COVAR	17379	0.0044433	0.0085035	0.012178	0.35761
Nerve	COVAR	17379	0.0030704	0.0061855	0.0098602	0.37775
Thyroid	COVAR	17379	0.0030396	0.0060249	0.010465	0.38574
Fallopian_Tube	COVAR	17379	0.0031458	0.0061084	0.013375	0.40703
Heart	COVAR	17379	0.0019607	0.003376	0.0092896	0.41642
Adipose_Tissue	COVAR	17379	0.0015844	0.003113	0.012715	0.45042
Kidney	COVAR	17379	0.0011001	0.0019371	0.0092826	0.45283
Uterus	COVAR	17379	0.00090143	0.0018381	0.011752	0.46943
Muscle	COVAR	17379	-0.0006022	-0.0011395	0.0066857	0.53588
Prostate	COVAR	17379	-0.0022732	-0.004317	0.013293	0.56789

Colon	COVAR	17379	-0.0032431	-0.0060757	0.015029	0.58542
Bladder	COVAR	17379	-0.0038286	-0.0074687	0.01545	0.59786
Skin	COVAR	17379	-0.0035979	-0.0068655	0.009788	0.6434
Pituitary	COVAR	17379	-0.0037126	-0.0069287	0.0080679	0.6773
Stomach	COVAR	17379	-0.0097726	-0.017768	0.012939	0.77496
Esophagus	COVAR	17379	-0.013347	-0.025163	0.016561	0.78986
Pancreas	COVAR	17379	-0.0075092	-0.012564	0.0083058	0.81702
Lung	COVAR	17379	-0.0096814	-0.018734	0.010152	0.82986
Testis	COVAR	17379	-0.0070801	-0.012236	0.0053725	0.90621
Ovary	COVAR	17379	-0.014286	-0.02898	0.010003	0.92336
Brain	COVAR	17379	-0.012314	-0.021407	0.006543	0.97007
**54 specific tissue types**						
**VARIABLE**	**TYPE**	**NGENES**	**BETA**	**BETA_STD**	**SE**	**P**
Spleen	COVAR	17379	0.022817	0.045244	0.0071165	0.00067421
Adrenal_Gland	COVAR	17379	0.020447	0.039497	0.0096957	0.017489
Small_Intestine_Terminal_Ileum	COVAR	17379	0.017005	0.031298	0.0092086	0.032408
Cervix_Endocervix	COVAR	17379	0.018657	0.036969	0.011081	0.046137
Artery_Aorta	COVAR	17379	0.014607	0.029827	0.0098319	0.068701
Cervix_Ectocervix	COVAR	17379	0.016823	0.032999	0.011551	0.072659
Breast_Mammary_Tissue	COVAR	17379	0.015433	0.029703	0.011934	0.097978
Colon_Transverse	COVAR	17379	0.014081	0.02591	0.010894	0.098093
Adipose_Subcutaneous	COVAR	17379	0.011788	0.023544	0.010192	0.12374

Minor_Salivary_Gland	COVAR	17379	0.010564	0.019675	0.0091812	0.12496
Vagina	COVAR	17379	0.011415	0.021845	0.010566	0.14001
Artery_Tibial	COVAR	17379	0.010022	0.020646	0.0095433	0.14684
Fallopian_Tube	COVAR	17379	0.011585	0.022496	0.011259	0.15175
Nerve_Tibial	COVAR	17379	0.0097031	0.019547	0.0095788	0.15554
Thyroid	COVAR	17379	0.0087683	0.01738	0.0093107	0.17317
Heart_Left_Ventricle	COVAR	17379	0.0079574	0.01329	0.0087953	0.18281
Uterus	COVAR	17379	0.0092241	0.018808	0.010512	0.19013
Kidney_Medulla	COVAR	17379	0.0076135	0.01409	0.0089939	0.19864
Whole_Blood	COVAR	17379	0.0045978	0.0083106	0.0054967	0.20145
Liver	COVAR	17379	0.0046786	0.0084286	0.0063534	0.23075
Bladder	COVAR	17379	0.0091491	0.017848	0.012447	0.23116
Artery_Coronary	COVAR	17379	0.0077754	0.01549	0.01132	0.24609
Prostate	COVAR	17379	0.0077308	0.014681	0.011467	0.2501
Kidney_Cortex	COVAR	17379	0.0058105	0.010218	0.0087896	0.25429
Heart_Atrial_Appendage	COVAR	17379	0.0056577	0.010164	0.0091555	0.26831
Skin_Not_Sun_Exposed_Suprapubic	COVAR	17379	0.0040355	0.0077879	0.0074949	0.29515
Adipose_Visceral_Omentum	COVAR	17379	0.0055954	0.010876	0.010535	0.29767
Skin_Sun_Exposed_Lower_leg	COVAR	17379	0.003514	0.0068238	0.0075104	0.31994
Muscle_Skeletal	COVAR	17379	0.0022352	0.0042293	0.0066542	0.36847
Esophagus_Mucosa	COVAR	17379	0.0021433	0.0041457	0.007436	0.38659
Esophagus_Gastroesophageal_Junction	COVAR	17379	0.0021661	0.0042672	0.012251	0.42983

Esophagus_Muscularis	COVAR	17379	0.0020081	0.0039638	0.011857	0.43276
Stomach	COVAR	17379	0.0013353	0.0024279	0.011361	0.45322
Pituitary	COVAR	17379	0.00052156	0.00097336	0.0086956	0.47609
Cells_EBV-transformed_lymphocytes	COVAR	17379	-1.52E-05	-3.31E-05	0.0047252	0.50128
Colon_Sigmoid	COVAR	17379	-0.0014316	-0.002803	0.012043	0.54731
Lung	COVAR	17379	-0.0016466	-0.0031862	0.0087493	0.57464
Cells_Cultured_fibroblasts	COVAR	17379	-0.0021556	-0.0046207	0.006276	0.63437
Pancreas	COVAR	17379	-0.0031812	-0.0053228	0.0079531	0.65542
Ovary	COVAR	17379	-0.0059092	-0.011987	0.0093488	0.73633
Brain_Cerebellar_Hemisphere	COVAR	17379	-0.0041315	-0.0082959	0.0058498	0.75998
Brain_Cerebellum	COVAR	17379	-0.0043824	-0.0087058	0.0060305	0.76629
Testis	COVAR	17379	-0.0057368	-0.0099149	0.0053545	0.858
Brain_Spinal_cord_cervical_c-1	COVAR	17379	-0.011259	-0.020253	0.0081156	0.91731
Brain_Substantia_nigra	COVAR	17379	-0.013926	-0.023993	0.0080388	0.95838
Brain_Cortex	COVAR	17379	-0.011904	-0.021726	0.0068031	0.95991
Brain_Putamen_basal_ganglia	COVAR	17379	-0.013378	-0.022782	0.0075176	0.96242
Brain_Frontal_Cortex_BA9	COVAR	17379	-0.012256	-0.022601	0.0065709	0.96891
Brain_Caudate_basal_ganglia	COVAR	17379	-0.014379	-0.024857	0.0074501	0.97319
Brain_Hypothalamus	COVAR	17379	-0.015262	-0.026296	0.0076462	0.97702
Brain_Nucleus_accumbens_basal_ganglia	COVAR	17379	-0.014603	-0.025357	0.0072325	0.97825
Brain_Anterior_cingulate_cortex_BA24	COVAR	17379	-0.013949	-0.024658	0.0068816	0.97866
Brain_Hippocampus	COVAR	17379	-0.015733	-0.026666	0.0075498	0.98141

Brain_Amygdala	COVAR	17379	-0.015647	-0.026678	0.0074274	0.98242

MAGMA gene-property analysis (FUMA) was used to test the association between gene-level GWAS signal and tissue-specific expression, using a one-sided test ($\beta>0$) and Bonferroni correction across tissues (30 general; 54 specific).

TYPE: denotes the variable type (SET = gene set; COVAR = gene covariate). **NGENES**: total genes

Supplementary Table 9: cis-eQTL analysis results

rsID	*uniqID*	*chr*	*pos*	*Tested Allele*	*DB*	*tissue*	*Gene*	*Symbol*	*P-value*	*FDR*	*signed_stats*	*RiskincAllele*	*alignedDirection*
rs6066146	20:45599553:A:G	20	45599553	G	GTEx/v7	Thyroid	ENSG00000064655	*EYA2*	1.454E-06	4.329E-15	0.245	A	-
rs13043230	20:45600617:C:T	20	45600617	C	GTEx/v7	Thyroid	ENSG00000064655	*EYA2*	9.054E-06	4.329E-15	-0.220	C	-
rs11699232	20:45604745:A:G	20	45604745	G	GTEx/v7	Thyroid	ENSG00000064655	*EYA2*	1.770E-05	4.329E-15	-0.216	G	-
rs35930260	20:45595465:C:CT	20	45595465	CT	GTEx/v7	Thyroid	ENSG00000064655	*EYA2*	1.774E-05	4.329E-15	-0.225	CT	-
rs13041611	20:45600309:C:T	20	45600309	T	GTEx/v7	Thyroid	ENSG00000064655	*EYA2*	2.066E-05	4.329E-15	-0.212	T	-
rs13042847	20:45602299:C:T	20	45602299	T	GTEx/v7	Thyroid	ENSG00000064655	*EYA2*	2.667E-05	4.329E-15	-0.210	T	-
rs71183224	20:45603386:C:CT	20	45603386	CT	GTEx/v7	Thyroid	ENSG00000064655	*EYA2*	0.000031	4.329E-15	-0.209	CT	-
rs6066146	20:45599553:A:G	20	45599553	G	GTEx/v8	Thyroid	ENSG00000064655	*EYA2*	0.000124	1.035E-20	0.169	A	-

cis-eQTL associations were retrieved via FUMA SNP2GENE using GTEx (v7/v8) resources. eQTL P values correspond to tests of association between genotype and normalized gene expression based on linear regression (two-sided). Multiple testing is reflected by the reported FDR from the original eQTL resources.

Chromatin interactions analysis results

GenomicLocus	*region1*	*region2*	*FDR*	*type*	*DB*	*tissue/cell*	*inter/intra*	*SNPs*	*Genes*
2	20:45560001-45600000	20:46880001-46920000	3.44419E-10	HiC	GSE87112	Mesendoderm	intra	rs7261550;rs7261621;rs35930260;rs35601418;rs11699842;rs6066146;rs13037317	nan
2	20:45560001-45600000	20:45360001-45400000	6.20606E-34	HiC	GSE87112	Mesendoderm	intra	rs7261550;rs7261621;rs35930260;rs35601418;rs11699842;rs6066146;rs13037317	nan
2	20:45560001-45600000	20:45320001-45360000	3.29387E-09	HiC	GSE87112	Mesendoderm	intra	rs7261550;rs7261621;rs35930260;rs35601418;rs11699842;rs6066146;rs13037317	ENSG00000197496
2	20:45600001-45640000	20:45120001-45160000	1.68552E-09	HiC	GSE87112	Mesendoderm	intra	rs13041757;rs13041611;rs13043230;rs11697925;rs13042954;rs13042847;rs13043269;rs71183224;rs13039645;rs11699232	ENSG00000198185
2	20:45560001-45600000	20:45080001-45120000	1.68552E-09	HiC	GSE87112	Mesendoderm	intra	rs7261550;rs7261621;rs35930260;rs35601418;rs11699842;rs6066146;rs13037317	nan

2	20:45560001-45600000	20:46120001-46160000	1.24105E-08	HiC	GSE87112	Mesendoderm	intra	rs7261550;rs7261621;rs35930260;rs35601418;rs11699842;rs6066146;rs13037317	ENSG00000124151
2	20:45560001-45600000	20:45760001-45800000	6.26684E-53	HiC	GSE87112	Mesendoderm	intra	rs7261550;rs7261621;rs35930260;rs35601418;rs11699842;rs6066146;rs13037317	nan
2	20:45560001-45600000	20:46960001-47000000	5.26838E-09	HiC	GSE87112	Mesendoderm	intra	rs7261550;rs7261621;rs35930260;rs35601418;rs11699842;rs6066146;rs13037317	nan
2	20:45560001-45600000	20:46200001-46240000	2.92972E-13	HiC	GSE87112	Mesendoderm	intra	rs7261550;rs7261621;rs35930260;rs35601418;rs11699842;rs6066146;rs13037317	nan
2	20:45560001-45600000	20:46240001-46280000	2.64239E-11	HiC	GSE87112	Mesendoderm	intra	rs7261550;rs7261621;rs35930260;rs35601418;rs11699842;rs6066146;rs13037317	nan
2	20:45600001-45640000	20:46240001-46280000	8.04219E-07	HiC	GSE87112	Mesendoderm	intra	rs13041757;rs13041611;rs13043230;rs11697925;rs13042954;rs13042847;rs13043269;rs71183224;rs13039645;rs11699232	nan
2	20:45600001-45640000	20:45760001-45800000	1.89172E-31	HiC	GSE87112	Mesendoderm	intra	rs13041757;rs13041611;rs13043230;rs11697925;rs13042954;rs13042847;rs13043269;rs71183224;rs13039645;rs11699232	nan
2	20:45600001-45640000	20:45720001-45760000	1.16632E-15	HiC	GSE87112	Mesendoderm	intra	rs13041757;rs13041611;rs13043230;rs11697925;rs13042954;rs13042847;rs13043269;rs71183224;rs13039645;rs11699232	nan
2	20:45600001-45640000	20:45680001-45720000	4.53332E-10	HiC	GSE87112	Mesendoderm	intra	rs13041757;rs13041611;rs13043230;rs11697925;rs13042954;rs13042847;rs13043269;rs71183224;rs13039645;rs11699232	nan
2	20:45560001-45600000	20:45720001-45760000	1.84048E-34	HiC	GSE87112	Mesendoderm	intra	rs7261550;rs7261621;rs35930260;rs35601418;rs11699842;rs6066146;rs13037317	nan
2	20:45560001-45600000	20:47000001-47040000	1.82425E-09	HiC	GSE87112	Mesendoderm	intra	rs7261550;rs7261621;rs35930260;rs35601418;rs11699842;rs6066146;rs13037317	nan
2	20:45560001-45600000	20:45680001-45720000	4.16537E-09	HiC	GSE87112	Trophoblast-like_Cell	intra	rs7261550;rs7261621;rs35930260;rs35601418;rs11699842;rs6066146;rs13037317	nan
2	20:45560001-45600000	20:45400001-45440000	1.45834E-47	HiC	GSE87112	Mesendoderm	intra	rs7261550;rs7261621;rs35930260;rs35601418;rs11699842;rs6066146;rs13037317	nan
2	20:45560001-45600000	20:45520001-45560000	3.62698E-18	HiC	GSE87112	hESC	intra	rs7261550;rs7261621;rs35930260;rs35601418;rs11699842;rs6066146;rs13037317	ENSG00000064655
2	20:45600001-45640000	20:45400001-45440000	5.79985E-25	HiC	GSE87112	hESC	intra	rs13041757;rs13041611;rs13043230;rs11697925;rs13042954;rs13042847;rs13043269;rs71183224;rs13039645;rs11699232	nan
2	20:45560001-45600000	20:45400001-45440000	6.11276E-37	HiC	GSE87112	hESC	intra	rs7261550;rs7261621;rs35930260;rs35601418;rs11699842;rs6066146;rs13037317	nan

2	20:45600001-45640000	20:45360001-45400000	6.03188E-35	HiC	GSE87112	hESC	intra	rs13041757;rs13041611;rs13043230;rs11697925;rs13042954;rs13042847;rs13043269;rs71183224;rs13039645;rs11699232	nan
2	20:45560001-45600000	20:45360001-45400000	9.57191E-31	HiC	GSE87112	hESC	intra	rs7261550;rs7261621;rs35930260;rs35601418;rs11699842;rs6066146;rs13037317	nan
2	20:45600001-45640000	20:45320001-45360000	2.12293E-07	HiC	GSE87112	hESC	intra	rs13041757;rs13041611;rs13043230;rs11697925;rs13042954;rs13042847;rs13043269;rs71183224;rs13039645;rs11699232	ENSG00000197496
2	20:45600001-45640000	20:46240001-46280000	2.42078E-08	HiC	GSE87112	hESC	intra	rs13041757;rs13041611;rs13043230;rs11697925;rs13042954;rs13042847;rs13043269;rs71183224;rs13039645;rs11699232	nan
2	20:45600001-45640000	20:45760001-45800000	8.0048E-22	HiC	GSE87112	hESC	intra	rs13041757;rs13041611;rs13043230;rs11697925;rs13042954;rs13042847;rs13043269;rs71183224;rs13039645;rs11699232	nan
2	20:45600001-45640000	20:45720001-45760000	8.23666E-10	HiC	GSE87112	hESC	intra	rs13041757;rs13041611;rs13043230;rs11697925;rs13042954;rs13042847;rs13043269;rs71183224;rs13039645;rs11699232	nan
2	20:45600001-45640000	20:45680001-45720000	6.67889E-09	HiC	GSE87112	hESC	intra	rs13041757;rs13041611;rs13043230;rs11697925;rs13042954;rs13042847;rs13043269;rs71183224;rs13039645;rs11699232	nan
2	20:45560001-45600000	20:47320001-47360000	9.12132E-07	HiC	GSE87112	hESC	intra	rs7261550;rs7261621;rs35930260;rs35601418;rs11699842;rs6066146;rs13037317	nan
2	20:45560001-45600000	20:45760001-45800000	8.62786E-49	HiC	GSE87112	hESC	intra	rs7261550;rs7261621;rs35930260;rs35601418;rs11699842;rs6066146;rs13037317	nan
2	20:45560001-45600000	20:45720001-45760000	6.13293E-19	HiC	GSE87112	hESC	intra	rs7261550;rs7261621;rs35930260;rs35601418;rs11699842;rs6066146;rs13037317	nan
2	20:45600001-45640000	20:45360001-45400000	1.14629E-49	HiC	GSE87112	Mesendoderm	intra	rs13041757;rs13041611;rs13043230;rs11697925;rs13042954;rs13042847;rs13043269;rs71183224;rs13039645;rs11699232	nan
2	20:45560001-45600000	20:45680001-45720000	2.97014E-24	HiC	GSE87112	hESC	intra	rs7261550;rs7261621;rs35930260;rs35601418;rs11699842;rs6066146;rs13037317	nan
2	20:45600001-45640000	20:45560001-45600000	2.3798E-08	HiC	GSE87112	Trophoblast-like_Cell	intra	rs13041757;rs13041611;rs13043230;rs11697925;rs13042954;rs13042847;rs13043269;rs71183224;rs13039645;rs11699232	nan
2	20:45560001-45600000	20:45520001-45560000	2.81391E-09	HiC	GSE87112	Trophoblast-like_Cell	intra	rs7261550;rs7261621;rs35930260;rs35601418;rs11699842;rs6066146;rs13037317	ENSG00000064655
2	20:45600001-45640000	20:45400001-45440000	2.44096E-20	HiC	GSE87112	Trophoblast-like_Cell	intra	rs13041757;rs13041611;rs13043230;rs11697925;rs13042954;rs13042847;rs13043269;rs71183224;rs13039645;rs11699232	nan
2	20:45560001-45600000	20:45400001-45440000	1.30078E-11	HiC	GSE87112	Trophoblast-like_Cell	intra	rs7261550;rs7261621;rs35930260;rs35601418;rs11699842;rs6066146;rs13037317	nan

2	20:45600001-45640000	20:45360001-45400000	1.25964E-25	HiC	GSE87112	Trophoblast-like_Cell	intra	rs13041757;rs13041611;rs13043230;rs11697925;rs13042954;rs13042847;rs13043269;rs71183224;rs13039645;rs11699232	nan
2	20:45560001-45600000	20:45360001-45400000	1.65209E-23	HiC	GSE87112	Trophoblast-like_Cell	intra	rs7261550;rs7261621;rs35930260;rs35601418;rs11699842;rs6066146;rs13037317	nan
2	20:45600001-45640000	20:45760001-45800000	2.59806E-10	HiC	GSE87112	Trophoblast-like_Cell	intra	rs13041757;rs13041611;rs13043230;rs11697925;rs13042954;rs13042847;rs13043269;rs71183224;rs13039645;rs11699232	nan
2	20:45560001-45600000	20:45760001-45800000	3.81547E-14	HiC	GSE87112	Trophoblast-like_Cell	intra	rs7261550;rs7261621;rs35930260;rs35601418;rs11699842;rs6066146;rs13037317	nan
2	20:45560001-45600000	20:45720001-45760000	4.20334E-07	HiC	GSE87112	Trophoblast-like_Cell	intra	rs7261550;rs7261621;rs35930260;rs35601418;rs11699842;rs6066146;rs13037317	nan
2	20:45560001-45600000	20:45600001-45640000	2.3798E-08	HiC	GSE87112	Trophoblast-like_Cell	intra	rs7261550;rs7261621;rs35930260;rs35601418;rs11699842;rs6066146;rs13037317	nan
2	20:45600001-45640000	20:45560001-45600000	2.40065E-31	HiC	GSE87112	Mesendoderm	intra	rs13041757;rs13041611;rs13043230;rs11697925;rs13042954;rs13042847;rs13043269;rs71183224;rs13039645;rs11699232	nan
2	20:45560001-45600000	20:45520001-45560000	2.272E-18	HiC	GSE87112	Mesendoderm	intra	rs7261550;rs7261621;rs35930260;rs35601418;rs11699842;rs6066146;rs13037317	ENSG00000064655
2	20:45600001-45640000	20:45400001-45440000	5.46733E-30	HiC	GSE87112	Mesendoderm	intra	rs13041757;rs13041611;rs13043230;rs11697925;rs13042954;rs13042847;rs13043269;rs71183224;rs13039645;rs11699232	nan
2	20:45560001-45600000	20:45600001-45640000	1.02811E-13	HiC	GSE87112	hESC	intra	rs7261550;rs7261621;rs35930260;rs35601418;rs11699842;rs6066146;rs13037317	nan
2	20:45560001-45600000	20:45680001-45720000	2.27221E-25	HiC	GSE87112	Mesendoderm	intra	rs7261550;rs7261621;rs35930260;rs35601418;rs11699842;rs6066146;rs13037317	nan
2	20:45600000-45610000	20:46080000-46090000	0	HiC	PsychENCODE	Promoter_anchored_loops	intra	rs13041757;rs13041611;rs13043230;rs11697925;rs13042954;rs13042847;rs13043269;rs71183224;rs13039645;rs11699232	nan
2	20:45560001-45600000	20:45600001-45640000	2.40065E-31	HiC	GSE87112	Mesendoderm	intra	rs7261550;rs7261621;rs35930260;rs35601418;rs11699842;rs6066146;rs13037317	nan
2	20:45600001-45640000	20:45760001-45800000	2.83629E-46	HiC	GSE87112	IMR90	intra	rs13041757;rs13041611;rs13043230;rs11697925;rs13042954;rs13042847;rs13043269;rs71183224;rs13039645;rs11699232	nan
2	20:45600001-45640000	20:45720001-45760000	3.15869E-25	HiC	GSE87112	IMR90	intra	rs13041757;rs13041611;rs13043230;rs11697925;rs13042954;rs13042847;rs13043269;rs71183224;rs13039645;rs11699232	nan
2	20:45600001-45640000	20:45680001-45720000	7.24161E-11	HiC	GSE87112	IMR90	intra	rs13041757;rs13041611;rs13043230;rs11697925;rs13042954;rs13042847;rs13043269;rs71183224;rs13039645;rs11699232	nan

2	20:45560001-45600000	20:46200001-46240000	2.67152E-38	HiC	GSE87112	IMR90	intra	rs7261550;rs7261621;rs35930260;rs35601418;rs11699842;rs6066146;rs13037317	nan
2	20:45560001-45600000	20:46160001-46200000	5.4942E-08	HiC	GSE87112	IMR90	intra	rs7261550;rs7261621;rs35930260;rs35601418;rs11699842;rs6066146;rs13037317	nan
2	20:45560001-45600000	20:46120001-46160000	4.59382E-17	HiC	GSE87112	IMR90	intra	rs7261550;rs7261621;rs35930260;rs35601418;rs11699842;rs6066146;rs13037317	ENSG00000124151
2	20:45560001-45600000	20:45960001-46000000	2.08922E-17	HiC	GSE87112	IMR90	intra	rs7261550;rs7261621;rs35930260;rs35601418;rs11699842;rs6066146;rs13037317	ENSG00000101040
2	20:45560001-45600000	20:45880001-45920000	8.14146E-14	HiC	GSE87112	IMR90	intra	rs7261550;rs7261621;rs35930260;rs35601418;rs11699842;rs6066146;rs13037317	nan
2	20:45560001-45600000	20:45840001-45880000	1.00454E-24	HiC	GSE87112	IMR90	intra	rs7261550;rs7261621;rs35930260;rs35601418;rs11699842;rs6066146;rs13037317	nan
2	20:45560001-45600000	20:45760001-45800000	1.4411E-78	HiC	GSE87112	IMR90	intra	rs7261550;rs7261621;rs35930260;rs35601418;rs11699842;rs6066146;rs13037317	nan
2	20:45560001-45600000	20:45720001-45760000	8.07357E-41	HiC	GSE87112	IMR90	intra	rs7261550;rs7261621;rs35930260;rs35601418;rs11699842;rs6066146;rs13037317	nan
2	20:45600001-45640000	20:46200001-46240000	4.3466E-12	HiC	GSE87112	IMR90	intra	rs13041757;rs13041611;rs13043230;rs11697925;rs13042954;rs13042847;rs13043269;rs71183224;rs13039645;rs11699232	nan
2	20:45560001-45600000	20:45680001-45720000	2.90385E-40	HiC	GSE87112	IMR90	intra	rs7261550;rs7261621;rs35930260;rs35601418;rs11699842;rs6066146;rs13037317	nan
2	20:45560001-45600000	20:45600001-45640000	5.22534E-29	HiC	GSE87112	IMR90	intra	rs7261550;rs7261621;rs35930260;rs35601418;rs11699842;rs6066146;rs13037317	nan
2	20:45600001-45640000	20:45560001-45600000	8.76112E-16	HiC	GSE87112	Spleen	intra	rs13041757;rs13041611;rs13043230;rs11697925;rs13042954;rs13042847;rs13043269;rs71183224;rs13039645;rs11699232	nan
2	20:45560001-45600000	20:45520001-45560000	1.50745E-09	HiC	GSE87112	Spleen	intra	rs7261550;rs7261621;rs35930260;rs35601418;rs11699842;rs6066146;rs13037317	ENSG00000064655
2	20:45560001-45600000	20:45600001-45640000	8.76112E-16	HiC	GSE87112	Spleen	intra	rs7261550;rs7261621;rs35930260;rs35601418;rs11699842;rs6066146;rs13037317	nan
2	20:45600001-45640000	20:45560001-45600000	2.851E-09	HiC	GSE87112	Liver	intra	rs13041757;rs13041611;rs13043230;rs11697925;rs13042954;rs13042847;rs13043269;rs71183224;rs13039645;rs11699232	nan
2	20:45600001-45640000	20:45400001-45440000	6.81445E-08	HiC	GSE87112	Liver	intra	rs13041757;rs13041611;rs13043230;rs11697925;rs13042954;rs13042847;rs13043269;rs71183224;rs13039645;rs11699232	nan

2	20:45560001-45600000	20:45600001-45640000	2.851E-09	HiC	GSE87112	Liver	intra	rs7261550;rs7261621;rs35930260;rs35601418;rs11699842;rs6066146;rs13037317	nan
2	20:45560001-45600000	20:46680001-46720000	6.14911E-11	HiC	GSE87112	Left_Ventricle	intra	rs7261550;rs7261621;rs35930260;rs35601418;rs11699842;rs6066146;rs13037317	nan
2	20:45560001-45600000	20:45720001-45760000	1.68622E-12	HiC	GSE87112	Left_Ventricle	intra	rs7261550;rs7261621;rs35930260;rs35601418;rs11699842;rs6066146;rs13037317	nan
2	20:45590000-45600000	20:46470000-46480000	0	HiC	PsychENCODE	Promoter_anchored_loops	intra	rs7261550;rs7261621;rs35930260;rs35601418;rs11699842;rs6066146;rs13037317	nan
2	20:45560001-45600000	20:45640001-45680000	7.02633E-08	HiC	GSE87112	IMR90	intra	rs7261550;rs7261621;rs35930260;rs35601418;rs11699842;rs6066146;rs13037317	nan
2	20:45600001-45640000	20:46240001-46280000	1.09036E-14	HiC	GSE87112	IMR90	intra	rs13041757;rs13041611;rs13043230;rs11697925;rs13042954;rs13042847;rs13043269;rs71183224;rs13039645;rs11699232	nan
2	20:45560001-45600000	20:45320001-45360000	2.64564E-18	HiC	GSE87112	IMR90	intra	rs7261550;rs7261621;rs35930260;rs35601418;rs11699842;rs6066146;rs13037317	ENSG00000197496
2	20:45600001-45640000	20:45320001-45360000	3.14643E-08	HiC	GSE87112	IMR90	intra	rs13041757;rs13041611;rs13043230;rs11697925;rs13042954;rs13042847;rs13043269;rs71183224;rs13039645;rs11699232	ENSG00000197496
2	20:45600001-45640000	20:45560001-45600000	6.08119E-21	HiC	GSE87112	Mesenchymal_Stem_Cell	intra	rs13041757;rs13041611;rs13043230;rs11697925;rs13042954;rs13042847;rs13043269;rs71183224;rs13039645;rs11699232	nan
2	20:45560001-45600000	20:45520001-45560000	7.97856E-17	HiC	GSE87112	Mesenchymal_Stem_Cell	intra	rs7261550;rs7261621;rs35930260;rs35601418;rs11699842;rs6066146;rs13037317	ENSG00000064655
2	20:45560001-45600000	20:45360001-45400000	6.47709E-08	HiC	GSE87112	Mesenchymal_Stem_Cell	intra	rs7261550;rs7261621;rs35930260;rs35601418;rs11699842;rs6066146;rs13037317	nan
2	20:45560001-45600000	20:45080001-45120000	3.79946E-08	HiC	GSE87112	Mesenchymal_Stem_Cell	intra	rs7261550;rs7261621;rs35930260;rs35601418;rs11699842;rs6066146;rs13037317	nan
2	20:45600001-45640000	20:45520001-45560000	1.15085E-07	HiC	GSE87112	hESC	intra	rs13041757;rs13041611;rs13043230;rs11697925;rs13042954;rs13042847;rs13043269;rs71183224;rs13039645;rs11699232	ENSG00000064655
2	20:45560001-45600000	20:47360001-47400000	1.69542E-09	HiC	GSE87112	Mesenchymal_Stem_Cell	intra	rs7261550;rs7261621;rs35930260;rs35601418;rs11699842;rs6066146;rs13037317	nan
2	20:45560001-45600000	20:47320001-47360000	3.33824E-08	HiC	GSE87112	Mesenchymal_Stem_Cell	intra	rs7261550;rs7261621;rs35930260;rs35601418;rs11699842;rs6066146;rs13037317	nan
2	20:45560001-45600000	20:47120001-47160000	7.83575E-09	HiC	GSE87112	Mesenchymal_Stem_Cell	intra	rs7261550;rs7261621;rs35930260;rs35601418;rs11699842;rs6066146;rs13037317	nan

2	20:45560001-45600000	20:47000001-47040000	9.42709E-09	HiC	GSE87112	Mesenchymal_Stem_Cell	intra	rs7261550;rs7261621;rs35930260;rs35601418;rs11699842;rs6066146;rs13037317	nan
2	20:45560001-45600000	20:46960001-47000000	6.62313E-12	HiC	GSE87112	Mesenchymal_Stem_Cell	intra	rs7261550;rs7261621;rs35930260;rs35601418;rs11699842;rs6066146;rs13037317	nan
2	20:45560001-45600000	20:46880001-46920000	6.68287E-11	HiC	GSE87112	Mesenchymal_Stem_Cell	intra	rs7261550;rs7261621;rs35930260;rs35601418;rs11699842;rs6066146;rs13037317	nan
2	20:45560001-45600000	20:46840001-46880000	9.32147E-07	HiC	GSE87112	Mesenchymal_Stem_Cell	intra	rs7261550;rs7261621;rs35930260;rs35601418;rs11699842;rs6066146;rs13037317	nan
2	20:45560001-45600000	20:46720001-46760000	3.44567E-13	HiC	GSE87112	Mesenchymal_Stem_Cell	intra	rs7261550;rs7261621;rs35930260;rs35601418;rs11699842;rs6066146;rs13037317	nan
2	20:45560001-45600000	20:45760001-45800000	2.65029E-09	HiC	GSE87112	Mesenchymal_Stem_Cell	intra	rs7261550;rs7261621;rs35930260;rs35601418;rs11699842;rs6066146;rs13037317	nan
2	20:45560001-45600000	20:45720001-45760000	2.42913E-10	HiC	GSE87112	Mesenchymal_Stem_Cell	intra	rs7261550;rs7261621;rs35930260;rs35601418;rs11699842;rs6066146;rs13037317	nan
2	20:45560001-45600000	20:45680001-45720000	6.13821E-15	HiC	GSE87112	Mesenchymal_Stem_Cell	intra	rs7261550;rs7261621;rs35930260;rs35601418;rs11699842;rs6066146;rs13037317	nan
2	20:45560001-45600000	20:45600001-45640000	6.08119E-21	HiC	GSE87112	Mesenchymal_Stem_Cell	intra	rs7261550;rs7261621;rs35930260;rs35601418;rs11699842;rs6066146;rs13037317	nan
2	20:45600001-45640000	20:45560001-45600000	5.22534E-29	HiC	GSE87112	IMR90	intra	rs13041757;rs13041611;rs13043230;rs11697925;rs13042954;rs13042847;rs13043269;rs71183224;rs13039645;rs11699232	nan
2	20:45560001-45600000	20:45520001-45560000	2.13245E-40	HiC	GSE87112	IMR90	intra	rs7261550;rs7261621;rs35930260;rs35601418;rs11699842;rs6066146;rs13037317	ENSG00000064655
2	20:45560001-45600000	20:45480001-45520000	7.24161E-11	HiC	GSE87112	IMR90	intra	rs7261550;rs7261621;rs35930260;rs35601418;rs11699842;rs6066146;rs13037317	nan
2	20:45560001-45600000	20:45440001-45480000	1.2422E-09	HiC	GSE87112	IMR90	intra	rs7261550;rs7261621;rs35930260;rs35601418;rs11699842;rs6066146;rs13037317	nan
2	20:45600001-45640000	20:45400001-45440000	5.51467E-36	HiC	GSE87112	IMR90	intra	rs13041757;rs13041611;rs13043230;rs11697925;rs13042954;rs13042847;rs13043269;rs71183224;rs13039645;rs11699232	nan
2	20:45560001-45600000	20:45400001-45440000	2.09192E-55	HiC	GSE87112	IMR90	intra	rs7261550;rs7261621;rs35930260;rs35601418;rs11699842;rs6066146;rs13037317	nan
2	20:45600001-45640000	20:45360001-45400000	4.23606E-52	HiC	GSE87112	IMR90	intra	rs13041757;rs13041611;rs13043230;rs11697925;rs13042954;rs13042847;rs13043269;rs71183224;rs13039645;rs11699232	nan

2	20:45560001-45600000	20:45360001-45400000	4.0312E-72	HiC	GSE87112	IMR90	intra	rs7261550;rs7261621;rs35930260;rs35601418;rs11699842;rs6066146;rs13037317	nan
2	20:45560001-45600000	20:45640001-45680000	2.70447E-07	HiC	GSE87112	Mesendoderm	intra	rs7261550;rs7261621;rs35930260;rs35601418;rs11699842;rs6066146;rs13037317	nan
2	20:45600001-45640000	20:45560001-45600000	1.02811E-13	HiC	GSE87112	hESC	intra	rs13041757;rs13041611;rs13043230;rs11697925;rs13042954;rs13042847;rs13043269;rs71183224;rs13039645;rs11699232	nan

Chromatin interaction links were obtained from the FUMA built-in Hi-C resources. Significant chromatin loops were defined based on FDR (default FDR ≤ 1 × 10^{-6}) prior to gene mapping.

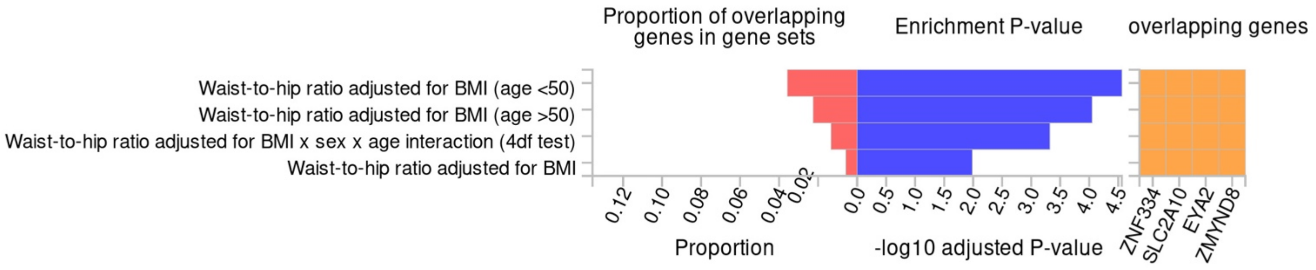

Supplementary Figure 2. Overrepresentation of mapping genes in GWAS Catalog Sets

The x-axis shows the proportion of overlapping genes and their enrichment p value, and the y-axis lists traits from the GWAS catalog. Overlapping genes are noted on the right.

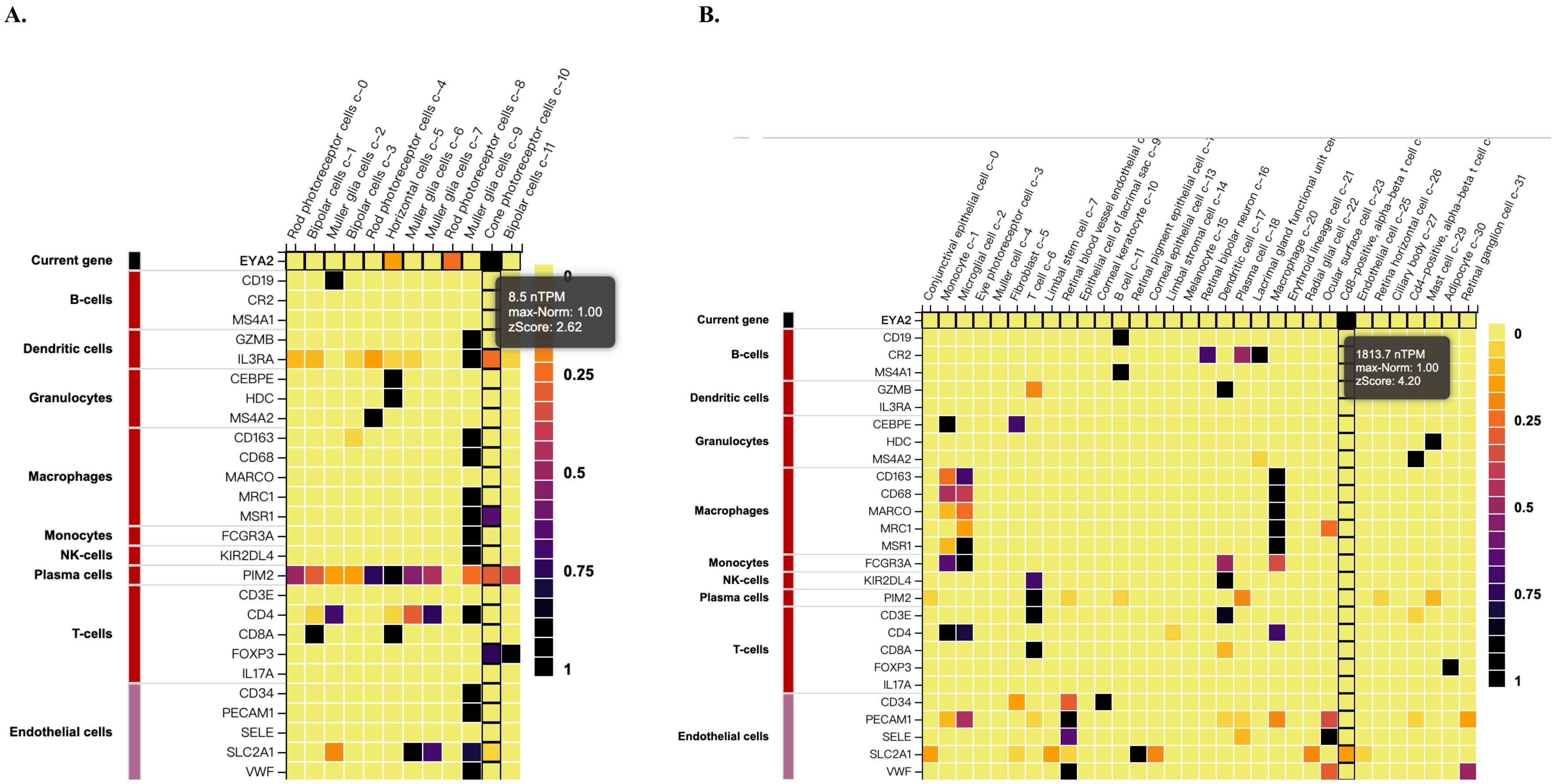
A.
Rod photoreceptor cells c–0
Bipolar cells c–1
Muller glia cells c–2
Bipolar cells c–3
Rod photoreceptor cells c–4
Horizontal cells c–5
Muller glia cells c–6
Muller glia cells c–7
Rod photoreceptor cells c–8
Muller glia cells c–9
Cone photoreceptor cells c–10
Bipolar cells c–11
Current gene
EYA2
B-cells
CD19
CR2
MS4A1
Dendritic cells
GZMB
IL3RA
Granulocytes
CEBPE
HDC
MS4A2
Macrophages
CD163
CD68
MARCO
MRC1
MSR1
Monocytes
FCGR3A
NK-cells
KIR2DL4
Plasma cells
PIM2
T-cells
CD3E
CD4
CD8A
FOXP3
IL17A
Endothelial cells
CD34
PECAM1
SELE
SLC2A1
VWF
8.5 nTPM
max-Norm: 1.00
zScore: 2.62
0
0.25
0.5
0.75
1
B.
Conjunctival epithelial cell c–0
Monocyte c–1
Microglial cell c–2
Eye photoreceptor cell c–3
Muller cell c–4
Fibroblast c–5
T cell c–6
Limbal stem cell c–7
Retinal blood vessel endothelial c
Epithelial cell of lacrimal sac c–9
Corneal keratocyte c–10
B cell c–11
Retinal pigment epithelial cell c–
Corneal epithelial cell c–13
Limbal stromal cell c–14
Melanocyte c–15
Retinal bipolar neuron c–16
Dendritic cell c–17
Plasma cell c–18
Lacrimal gland functional unit cel
Macrophage c–20
Erythroid lineage cell c–21
Radial glial cell c–22
Ocular surface cell c–23
Cd8–positive, alpha–beta t cell c
Endothelial cell c–25
Retina horizontal cell c–26
Ciliary body c–27
Cd4–positive, alpha–beta t cell c
Mast cell c–29
Adipocyte c–30
Retinal ganglion cell c–31
Current gene
EYA2
B-cells
CD19
CR2
MS4A1
Dendritic cells
GZMB
IL3RA
Granulocytes
CEBPE
HDC
MS4A2
Macrophages
CD163
CD68
MARCO
MRC1
MSR1
Monocytes
FCGR3A
NK-cells
KIR2DL4
Plasma cells
PIM2
T-cells
CD3E
CD4
CD8A
FOXP3
IL17A
Endothelial cells
CD34
PECAM1
SELE
SLC2A1
VWF
1813.7 nTPM
max-Norm: 1.00
zScore: 4.20
0
0.25
0.5
0.75
1

C.

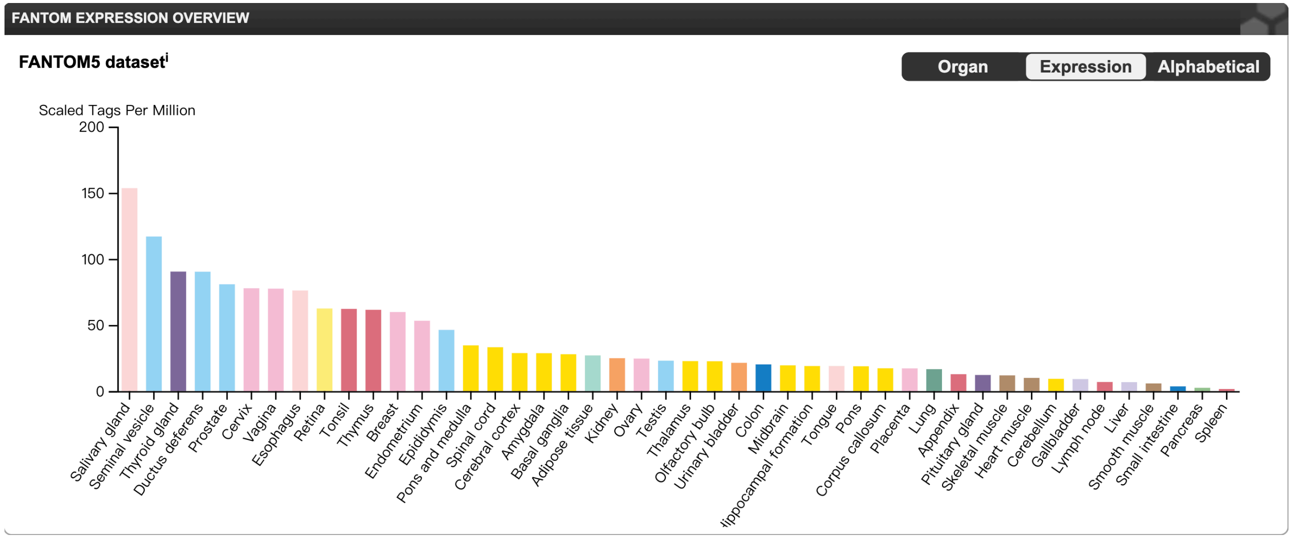

Supplementary Figure 3. Expression Profiles of *EYA2* Across Cell Types and Tissues

Panel (A): Heatmap displays *EYA2* expression profiles across various cell types identified in a single cell type dataset specific to ocular cells. Color intensity ranges from yellow (lower expression) to black (higher expression), representing normalized expression levels measured in normalized transcripts per million (nTPM). Key cell types highlighted include cone photoreceptor cells, rod photoreceptor cells, and horizontal cells among others.

Panel (B): Heatmap illustrates *EYA2* expression profiles from the Tabula Sapiens project, encompassing a broader spectrum of cell types beyond the ocular system. The expression levels across diverse cell types such as T cells and macrophages are presented, using the same color coding as Panel (A) to indicate expression intensity in nTPM.

Panel (C):A bar chart based on FANTOM5 data, showcasing the expression of *EYA2* across various human and animal organs and tissues. The expression levels are measured in scaled tags per million (TPM), with the retina shown to have one of the highest expressions, emphasizing the gene's significant presence in ocular tissues.

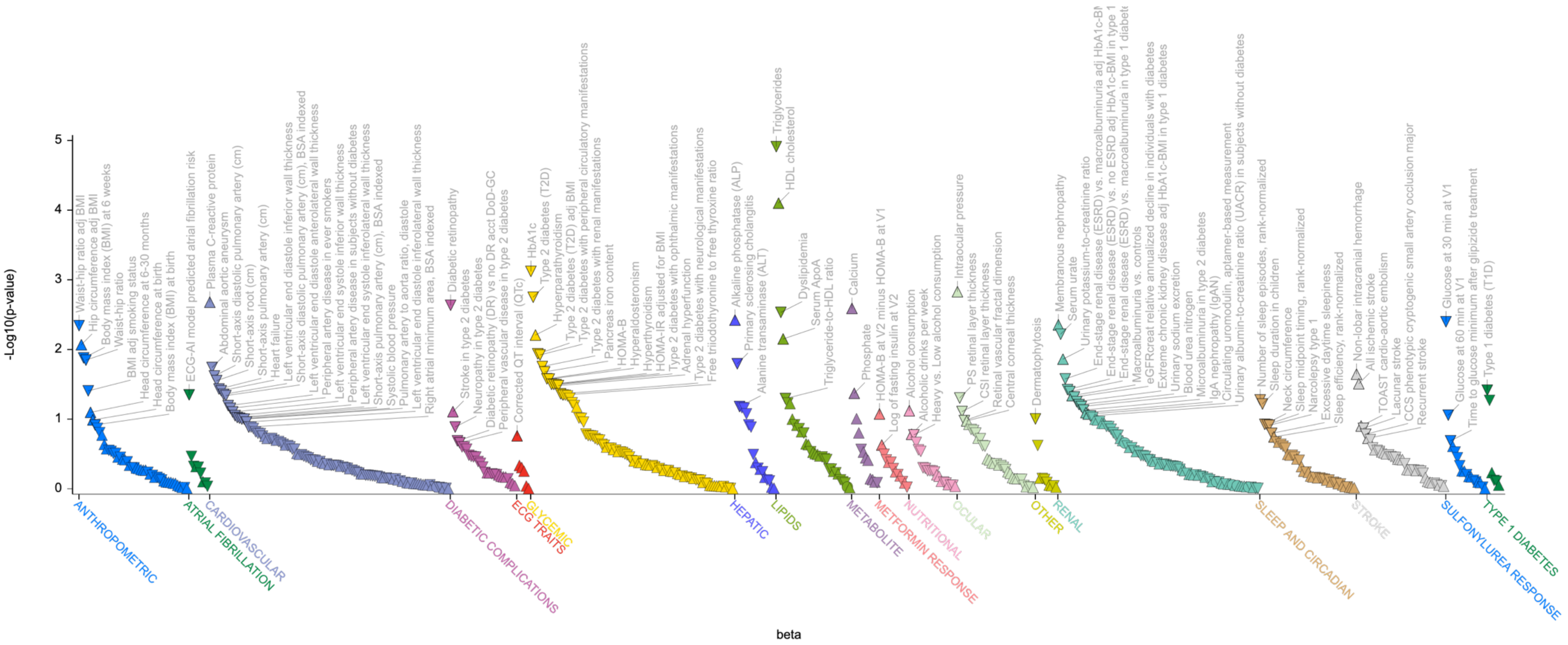

Supplementary Figure 4. Phenome-Wide Association Study of rs6066146

Nominal -log$_{10}$(p) for rs6066146 associations across trait categories, with upward/downward triangles indicating positive/negative effect sizes. The top signals include intraocular pressure, T2D, DR, triglycerides, and HDL cholesterol.

Supplementary Table 10: Phenome-wide association study Associations for rs6066146 with p < 0.05					
**chr:pos**	**phenotype**	**pValue**	**beta**	**standard errors**	**n**
20:45599553:A:G	description:"Triglycerides"	0.00001229	-0.0032	0.0006	2949190
20:45599553:A:G	description:"HDL cholesterol"	0.00007944	0.0024	0.0007	2557390
20:45599553:A:G	description:"HbA1c"	0.0007575	-0.0019	0.0004	1853380
20:45599553:A:G	description:"Intraocular pressure"	0.001483	0.0132	0.0042	169133
20:45599553:A:G	description:"Type 2 diabetes (T2D)"	0.00178	-0.0089	0.0016	3870720
20:45599553:A:G	description:"Plasma C-reactive protein"	0.002097	0.0039	0.0011	1290540
20:45599553:A:G	description:"Diabetic retinopathy"	0.002321	-0.0233	0.0074	183524
20:45599553:A:G	description:"Calcium"	0.002575	0.001	0.0004	1211010
20:45599553:A:G	description:"Dyslipidemia"	0.002937	-0.038	0.0128	56375
20:45599553:A:G	description:"Alkaline phosphatase (ALP)"	0.003785	0.0052	0.0013	1000830
20:45599553:A:G	description:"Glucose at 30 min at V1"	0.00400613	-0.1431068	0.049609872	832
20:45599553:A:G	description:"Membranous nephropathy"	0.004436	0.0755	0.0266	14853.3
20:45599553:A:G	description:"Waist-hip ratio adj BMI"	0.004555	-0.0057	0.0011	1666660
20:45599553:A:G	description:"Serum urate"	0.00604	-0.0051	0.0014	839200
20:45599553:A:G	description:"Hyperparathyroidism"	0.006211	0.04761585	0.017400727	32601.9
20:45599553:A:G	description:"Serum ApoA"	0.007064	0.005	0.0018	398828
20:45599553:A:G	description:"Hip circumference adj BMI"	0.008549	0.0116	0.0027	373332
20:45599553:A:G	description:"Type 2 diabetes (T2D) adj BMI"	0.01155	-0.0177	0.006	285309
20:45599553:A:G	description:"Type 2 diabetes with peripheral circulatory manifestations"	0.01199	-0.0344014	0.013692445	55714.3
20:45599553:A:G	description:"Body mass index (BMI) at 6 weeks"	0.01325	-0.0441	0.0178	6450
20:45599553:A:G	description:"Urinary potassium-to-creatinine ratio"	0.01377	0.0056	0.0023	327613
20:45599553:A:G	description:"Waist-hip ratio"	0.01421	-0.0053	0.001	2140730
20:45599553:A:G	description:"Primary sclerosing cholangitis"	0.01606	-0.0733	0.031	9269.73

20:45599553:A:G	description:"Type 2 diabetes with renal manifestations"	0.01757	-0.0217615	0.009164441	147098
20:45599553:A:G	description:"Abdominal aortic aneurysm"	0.01818	-0.0026468	0.001124906	1125328
20:45599553:A:G	description:"Pancreas iron content"	0.021	-0.02	0.0087	25617
20:45599553:A:G	description:"Non-lobar intracranial hemorrhage"	0.02265	0.1398	0.061334964	2253
20:45599553:A:G	description:"Short-axis diastolic pulmonary artery (cm)"	0.024	-0.0168	0.0071	41135
20:45599553:A:G	description:"HOMA-B"	0.02643	-0.0069	0.003	75033
20:45599553:A:G	description:"End-stage renal disease (ESRD) vs. macroalbuminuria adj HbA1c-BMI in type 1 diabetes"	0.02645	-0.1374	0.0619	4853
20:45599553:A:G	description:"Hyperaldosteronism"	0.0266	-0.0944007	0.042573877	5538.55
20:45599553:A:G	description:"Short-axis root (cm)"	0.028	-0.0136	0.0061	41135
20:45599553:A:G	description:"All ischemic stroke"	0.03106	0.0025	0.0024	1068480
20:45599553:A:G	description:"Hyperthyroidism"	0.03158	-0.0244	0.0113	26084.1
20:45599553:A:G	description:"HOMA-IR adjusted for BMI"	0.0317	-0.057	0.0265	2170
20:45599553:A:G	description:"Type 2 diabetes with ophthalmic manifestations"	0.03197	-0.0188218	0.008775588	169115
20:45599553:A:G	description:"Adrenal hyperfunction"	0.03302	-0.0834216	0.03913127	6747.99
20:45599553:A:G	description:"Short-axis pulmonary artery (cm)"	0.037	-0.0145	0.007	41135
20:45599553:A:G	description:"End-stage renal disease (ESRD) vs. no ESRD adj HbA1c-BMI in type 1 diabetes"	0.03773	-0.0909	0.0437	7762
20:45599553:A:G	description:"Type 1 diabetes (T1D)"	0.03845	-0.0153	0.007	229743
20:45599553:A:G	description:"Heart failure"	0.0392	-0.0091	0.0032	1063570
20:45599553:A:G	description:"BMI adj smoking status"	0.03928	-0.008	0.0027	328781
20:45599553:A:G	description:"End-stage renal disease (ESRD) vs. macroalbuminuria in type 1 diabetes"	0.04028	-0.1053	0.0513	4853
20:45599553:A:G	description:"Phosphate"	0.04259	0.0042	0.0021	400471
20:45599553:A:G	description:"Type 2 diabetes with neurological manifestations"	0.04332	-0.0148886	0.00736837	288946
20:45599553:A:G	description:"Left ventricular end diastole inferior wall thickness"	0.044	-0.0139	0.0069	42122
20:45599553:A:G	description:"ECG-AI model predicted atrial fibrillation risk"	0.04489	-0.0123	0.0062	39986

20:45599553:A:G	description:"Free triiodothyronine to free thyroxine ratio"	0.04515	-0.0137	0.0065	44161
20:45599553:A:G	description:"Fasting insulin"	0.04531	-0.0058	0.0016	221738
20:45599553:A:G	description:"Macroalbuminuria vs. controls"	0.04597	0.1017	0.0508	300
20:45599553:A:G	description:"Fasting proinsulin"	0.04652	-0.0118	0.0048	56562
20:45599553:A:G	description:"eGFRcreat relative annualized decline in individuals with diabetes"	0.04821	-0.0109161	0.006064522	46424
20:45599553:A:G	description:"PS retinal layer thickness"	0.04966	-0.0146	0.0075	38485

Supplementary Table 11: Transcriptome-Wide Association Study Results Across Tissues

Gene	CHR	HSQ	BEST.GWAS.ID	BEST.GWAS.Z	EQTL.ID	EQTL.R2	EQTL.Z	EQTL.GWAS.Z	NSNP	NWGT	MODEL	MODELCV.R2	MODELCV.PV	TWAS.Z	TWAS.P	Tissue
EYA2	20	0.31	rs6066146	5.5	rs6125025	4.66E-02	4.13	1.99	593	15	enet	0.09	2.40E-03	2.19	0.0282	Spleen
EYA2	20	0.29	rs6066146	5.5	rs4809607	1.59E-01	6.95	-2.10	593	1	top1	0.16	3.30E-12	-2.10	0.0357	Thyroid

HSQ: Heritability of the gene; ***BEST.GWAS.ID***: rsID of the most significant GWAS SNP in locus; ***BEST.GWAS.Z****:* Z-score of the most significant GWAS SNP in locus; ***EQTL.ID****:* rsID of the best eQTL in the locus; ***EQTL.R2****:* cross-validation R2 of the best eQTL in the locus; ***EQTL.Z****:* Z-score of the best eQTL in the locus; ***EQTL.GWAS.Z****:* GWAS Z-score for this eQTL; ***NSNP****:* Number of SNPs in the locus; ***NWGT****:* Number of expression weights; ***MODEL****:* Best performing model; ***MODELCV.R2****:* cross-validation R2 of the best performing model; ***MODELCV.PV***: cross-validation P-value of the best performing model; ***TWAS.Z****:* TWAS Z-score (our primary statistic of interest); ***TWAS.P****:* TWAS P-value.
top1: Single best eQTL; ***enet:*** *Elastic-net regression (with mixing parameter of 0.5).*

Supplementary Table 12: Mendelian Randomization Analysis of Four Disorders on Diabetic Retinopathy						
Type 2 diabetes (adjusted for BMI)						
**exposure**	**outcome**	**method**	**nsnp**	**b**	**se**	**pval**
Type 2 diabetes (adjusted for BMI) || id:ebi-a-GCST007516	DR	Inverse variance weighted	56	0.03503	0.0078	7E-06
Type 2 diabetes (adjusted for BMI) || id:ebi-a-GCST007516	DR	MR Egger	56	0.0387	0.0168	0.025
Type 2 diabetes (adjusted for BMI) || id:ebi-a-GCST007516	DR	Weighted median	56	0.02997	0.0123	0.015
Type 2 diabetes (adjusted for BMI) || id:ebi-a-GCST007516	DR	Simple median	56	0.03042	0.0114	0.008
Type 2 diabetes (adjusted for BMI) || id:ebi-a-GCST007516	DR	Weighted mode	56	0.03189	0.0116	0.008
**heterogeneity statistics results**						
**exposure**	**outcome**	**method**	**Q**	**Q_df**	**Q_pval**	
Type 2 diabetes (adjusted for BMI) || id:ebi-a-GCST007516	DR	Inverse variance weighted	64.83993475	55	0.171	
Type 2 diabetes (adjusted for BMI) || id:ebi-a-GCST007516	DR	MR Egger	64.76634336	54	0.1498	
**Horizontal pleiotropy results**						
**exposure**	**outcome**	**egger_intercept**	**se**	**pval**		
Type 2 diabetes (adjusted for BMI) || id:ebi-a-GCST007516	DR	-0.000294173	0.001187592	0.8053		
**Single SNP analysis results**						
**exposure**	**outcome**	**samplesize**	**SNP**	**b**	**se**	**p**
Type 2 diabetes (adjusted for BMI) || id:ebi-a-GCST007516	DR	16988	rs10244051	-0.1505	0.0699	0.031
Type 2 diabetes (adjusted for BMI) || id:ebi-a-GCST007516	DR	16988	rs10758593	0.06409	0.0853	0.452
Type 2 diabetes (adjusted for BMI) || id:ebi-a-GCST007516	DR	16988	rs1077394	-0.0131	0.0969	0.893
Type 2 diabetes (adjusted for BMI) || id:ebi-a-GCST007516	DR	16988	rs10830963	0.00417	0.0439	0.924
Type 2 diabetes (adjusted for BMI) || id:ebi-a-GCST007516	DR	16988	rs10842994	-0.0133	0.0738	0.857
Type 2 diabetes (adjusted for BMI) || id:ebi-a-GCST007516	DR	16988	rs10906115	0.15595	0.1	0.119
Type 2 diabetes (adjusted for BMI) || id:ebi-a-GCST007516	DR	16988	rs10965250	0.04985	0.0356	0.162

Type 2 diabetes (adjusted for BMI) || id:ebi-a-GCST007516	DR	16988	rs11063069	0.10265	0.0866	0.236
Type 2 diabetes (adjusted for BMI) || id:ebi-a-GCST007516	DR	16988	rs11603334	-0.0482	0.0543	0.375
Type 2 diabetes (adjusted for BMI) || id:ebi-a-GCST007516	DR	16988	rs11708067	0.02045	0.0467	0.662
Type 2 diabetes (adjusted for BMI) || id:ebi-a-GCST007516	DR	16988	rs12571751	0.05014	0.0591	0.396
Type 2 diabetes (adjusted for BMI) || id:ebi-a-GCST007516	DR	16988	rs1260326	0.10102	0.0627	0.107
Type 2 diabetes (adjusted for BMI) || id:ebi-a-GCST007516	DR	16988	rs13133548	-0.0815	0.0994	0.413
Type 2 diabetes (adjusted for BMI) || id:ebi-a-GCST007516	DR	16988	rs13266634	-0.0081	0.041	0.844
Type 2 diabetes (adjusted for BMI) || id:ebi-a-GCST007516	DR	16988	rs13389219	0.01867	0.0476	0.695
Type 2 diabetes (adjusted for BMI) || id:ebi-a-GCST007516	DR	16988	rs1359790	0.00573	0.064	0.929
Type 2 diabetes (adjusted for BMI) || id:ebi-a-GCST007516	DR	16988	rs1531343	0.00913	0.078	0.907
Type 2 diabetes (adjusted for BMI) || id:ebi-a-GCST007516	DR	16988	rs16826069	0.0418	0.0857	0.626
Type 2 diabetes (adjusted for BMI) || id:ebi-a-GCST007516	DR	16988	rs1801212	0.07656	0.0596	0.199
Type 2 diabetes (adjusted for BMI) || id:ebi-a-GCST007516	DR	16988	rs1801282	0.10349	0.0576	0.072
Type 2 diabetes (adjusted for BMI) || id:ebi-a-GCST007516	DR	16988	rs2237895	0.06497	0.0418	0.12
Type 2 diabetes (adjusted for BMI) || id:ebi-a-GCST007516	DR	16988	rs2395163	-0.0961	0.0806	0.233
Type 2 diabetes (adjusted for BMI) || id:ebi-a-GCST007516	DR	16988	rs243021	0.13362	0.0823	0.105
Type 2 diabetes (adjusted for BMI) || id:ebi-a-GCST007516	DR	16988	rs2796441	0.07537	0.0838	0.368
Type 2 diabetes (adjusted for BMI) || id:ebi-a-GCST007516	DR	16988	rs28265	0.16569	0.0657	0.012
Type 2 diabetes (adjusted for BMI) || id:ebi-a-GCST007516	DR	16988	rs2943641	-0.0164	0.0584	0.778
Type 2 diabetes (adjusted for BMI) || id:ebi-a-GCST007516	DR	16988	rs340874	0.17453	0.0691	0.012
Type 2 diabetes (adjusted for BMI) || id:ebi-a-GCST007516	DR	16988	rs35658696	0.07898	0.0564	0.161
Type 2 diabetes (adjusted for BMI) || id:ebi-a-GCST007516	DR	16988	rs35720761	0.12256	0.0717	0.087
Type 2 diabetes (adjusted for BMI) || id:ebi-a-GCST007516	DR	16988	rs4402960	0.0533	0.038	0.161
Type 2 diabetes (adjusted for BMI) || id:ebi-a-GCST007516	DR	16988	rs4457053	0.0193	0.075	0.797
Type 2 diabetes (adjusted for BMI) || id:ebi-a-GCST007516	DR	16988	rs4502156	0.1498	0.0836	0.073

Type 2 diabetes (adjusted for BMI) || id:ebi-a-GCST007516	DR	16988	rs459193	0.00227	0.0724	0.975
Type 2 diabetes (adjusted for BMI) || id:ebi-a-GCST007516	DR	16988	rs4607103	0.02177	0.0936	0.816
Type 2 diabetes (adjusted for BMI) || id:ebi-a-GCST007516	DR	16988	rs4812831	0.10126	0.097	0.297
Type 2 diabetes (adjusted for BMI) || id:ebi-a-GCST007516	DR	16988	rs5015480	0.08419	0.0482	0.081
Type 2 diabetes (adjusted for BMI) || id:ebi-a-GCST007516	DR	16988	rs516946	0.07139	0.0609	0.241
Type 2 diabetes (adjusted for BMI) || id:ebi-a-GCST007516	DR	16988	rs5219	0.03109	0.0544	0.567
Type 2 diabetes (adjusted for BMI) || id:ebi-a-GCST007516	DR	16988	rs55834942	-0.0514	0.083	0.536
Type 2 diabetes (adjusted for BMI) || id:ebi-a-GCST007516	DR	16988	rs58542926	0.0069	0.0712	0.923
Type 2 diabetes (adjusted for BMI) || id:ebi-a-GCST007516	DR	16988	rs60980157	0.02919	0.0659	0.658
Type 2 diabetes (adjusted for BMI) || id:ebi-a-GCST007516	DR	16988	rs6905288	-0.0639	0.0833	0.443
Type 2 diabetes (adjusted for BMI) || id:ebi-a-GCST007516	DR	16988	rs7177055	0.23479	0.0709	9E-04
Type 2 diabetes (adjusted for BMI) || id:ebi-a-GCST007516	DR	16988	rs7202877	-0.0565	0.0826	0.494
Type 2 diabetes (adjusted for BMI) || id:ebi-a-GCST007516	DR	16988	rs730497	-0.1165	0.1024	0.256
Type 2 diabetes (adjusted for BMI) || id:ebi-a-GCST007516	DR	16988	rs731839	-0.1497	0.0785	0.056
Type 2 diabetes (adjusted for BMI) || id:ebi-a-GCST007516	DR	16988	rs738409	-0.0445	0.0881	0.614
Type 2 diabetes (adjusted for BMI) || id:ebi-a-GCST007516	DR	16988	rs7501939	0.10214	0.0537	0.057
Type 2 diabetes (adjusted for BMI) || id:ebi-a-GCST007516	DR	16988	rs7756992	0.02005	0.0335	0.549
Type 2 diabetes (adjusted for BMI) || id:ebi-a-GCST007516	DR	16988	rs7903146	0.02974	0.0144	0.039
Type 2 diabetes (adjusted for BMI) || id:ebi-a-GCST007516	DR	16988	rs8042680	-0.0772	0.0831	0.353
Type 2 diabetes (adjusted for BMI) || id:ebi-a-GCST007516	DR	16988	rs8108269	0.07666	0.0664	0.248
Type 2 diabetes (adjusted for BMI) || id:ebi-a-GCST007516	DR	16988	rs864745	0.06667	0.0471	0.157
Type 2 diabetes (adjusted for BMI) || id:ebi-a-GCST007516	DR	16988	rs9379084	0.03939	0.0598	0.51
Type 2 diabetes (adjusted for BMI) || id:ebi-a-GCST007516	DR	16988	rs9388489	0.05439	0.0956	0.569
Type 2 diabetes (adjusted for BMI) || id:ebi-a-GCST007516	DR	16988	rs972283	-0.0292	0.0703	0.678

Type 2 diabetes (adjusted for BMI) || id:ebi-a-GCST007516	DR	16988	All - Inverse variance weighted	0.03503	0.0078	7E-06
Type 2 diabetes (adjusted for BMI) || id:ebi-a-GCST007516	DR	16988	All - MR Egger	0.0387	0.0168	0.025
**Leave-one-out analysis results**						
**exposure**	**outcome**	**samplesize**	**SNP**	**b**	**se**	**p**
Type 2 diabetes (adjusted for BMI) || id:ebi-a-GCST007516	DR	16988	rs10244051	0.037	0.0075	7E-07
Type 2 diabetes (adjusted for BMI) || id:ebi-a-GCST007516	DR	16988	rs10758593	0.03482	0.0079	1E-05
Type 2 diabetes (adjusted for BMI) || id:ebi-a-GCST007516	DR	16988	rs1077394	0.03529	0.0079	7E-06
Type 2 diabetes (adjusted for BMI) || id:ebi-a-GCST007516	DR	16988	rs10830963	0.03587	0.0079	6E-06
Type 2 diabetes (adjusted for BMI) || id:ebi-a-GCST007516	DR	16988	rs10842994	0.03549	0.0079	7E-06
Type 2 diabetes (adjusted for BMI) || id:ebi-a-GCST007516	DR	16988	rs10906115	0.0344	0.0078	1E-05
Type 2 diabetes (adjusted for BMI) || id:ebi-a-GCST007516	DR	16988	rs10965250	0.0344	0.008	2E-05
Type 2 diabetes (adjusted for BMI) || id:ebi-a-GCST007516	DR	16988	rs11063069	0.03456	0.0078	1E-05
Type 2 diabetes (adjusted for BMI) || id:ebi-a-GCST007516	DR	16988	rs11603334	0.0365	0.0078	3E-06
Type 2 diabetes (adjusted for BMI) || id:ebi-a-GCST007516	DR	16988	rs11708067	0.03538	0.0079	9E-06
Type 2 diabetes (adjusted for BMI) || id:ebi-a-GCST007516	DR	16988	rs12571751	0.0348	0.0079	1E-05
Type 2 diabetes (adjusted for BMI) || id:ebi-a-GCST007516	DR	16988	rs1260326	0.03415	0.0078	1E-05
Type 2 diabetes (adjusted for BMI) || id:ebi-a-GCST007516	DR	16988	rs13133548	0.03564	0.0078	5E-06
Type 2 diabetes (adjusted for BMI) || id:ebi-a-GCST007516	DR	16988	rs13266634	0.03639	0.0079	4E-06
Type 2 diabetes (adjusted for BMI) || id:ebi-a-GCST007516	DR	16988	rs13389219	0.03541	0.0079	8E-06
Type 2 diabetes (adjusted for BMI) || id:ebi-a-GCST007516	DR	16988	rs1359790	0.0354	0.0079	7E-06
Type 2 diabetes (adjusted for BMI) || id:ebi-a-GCST007516	DR	16988	rs1531343	0.03525	0.0079	8E-06
Type 2 diabetes (adjusted for BMI) || id:ebi-a-GCST007516	DR	16988	rs16826069	0.03498	0.0079	9E-06
Type 2 diabetes (adjusted for BMI) || id:ebi-a-GCST007516	DR	16988	rs1801212	0.03441	0.0079	1E-05
Type 2 diabetes (adjusted for BMI) || id:ebi-a-GCST007516	DR	16988	rs1801282	0.03395	0.0078	1E-05

Type 2 diabetes (adjusted for BMI) || id:ebi-a-GCST007516	DR	16988	rs2237895	0.03412	0.0079	2E-05
Type 2 diabetes (adjusted for BMI) || id:ebi-a-GCST007516	DR	16988	rs2395163	0.03607	0.0077	3E-06
Type 2 diabetes (adjusted for BMI) || id:ebi-a-GCST007516	DR	16988	rs243021	0.03427	0.0078	1E-05
Type 2 diabetes (adjusted for BMI) || id:ebi-a-GCST007516	DR	16988	rs2796441	0.03473	0.0079	1E-05
Type 2 diabetes (adjusted for BMI) || id:ebi-a-GCST007516	DR	16988	rs28265	0.03345	0.0077	1E-05
Type 2 diabetes (adjusted for BMI) || id:ebi-a-GCST007516	DR	16988	rs2943641	0.03581	0.0079	5E-06
Type 2 diabetes (adjusted for BMI) || id:ebi-a-GCST007516	DR	16988	rs340874	0.03351	0.0076	1E-05
Type 2 diabetes (adjusted for BMI) || id:ebi-a-GCST007516	DR	16988	rs35658696	0.0343	0.0079	1E-05
Type 2 diabetes (adjusted for BMI) || id:ebi-a-GCST007516	DR	16988	rs35720761	0.03414	0.0078	1E-05
Type 2 diabetes (adjusted for BMI) || id:ebi-a-GCST007516	DR	16988	rs4402960	0.03435	0.008	2E-05
Type 2 diabetes (adjusted for BMI) || id:ebi-a-GCST007516	DR	16988	rs4457053	0.03517	0.0079	8E-06
Type 2 diabetes (adjusted for BMI) || id:ebi-a-GCST007516	DR	16988	rs4502156	0.03417	0.0078	1E-05
Type 2 diabetes (adjusted for BMI) || id:ebi-a-GCST007516	DR	16988	rs459193	0.03535	0.0079	7E-06
Type 2 diabetes (adjusted for BMI) || id:ebi-a-GCST007516	DR	16988	rs4607103	0.0351	0.0079	8E-06
Type 2 diabetes (adjusted for BMI) || id:ebi-a-GCST007516	DR	16988	rs4812831	0.03466	0.0079	1E-05
Type 2 diabetes (adjusted for BMI) || id:ebi-a-GCST007516	DR	16988	rs5015480	0.03391	0.0079	2E-05
Type 2 diabetes (adjusted for BMI) || id:ebi-a-GCST007516	DR	16988	rs516946	0.03452	0.0079	1E-05
Type 2 diabetes (adjusted for BMI) || id:ebi-a-GCST007516	DR	16988	rs5219	0.0351	0.0079	1E-05
Type 2 diabetes (adjusted for BMI) || id:ebi-a-GCST007516	DR	16988	rs55834942	0.03568	0.0078	5E-06
Type 2 diabetes (adjusted for BMI) || id:ebi-a-GCST007516	DR	16988	rs58542926	0.03531	0.0079	8E-06
Type 2 diabetes (adjusted for BMI) || id:ebi-a-GCST007516	DR	16988	rs60980157	0.0351	0.0079	9E-06
Type 2 diabetes (adjusted for BMI) || id:ebi-a-GCST007516	DR	16988	rs6905288	0.03577	0.0078	5E-06
Type 2 diabetes (adjusted for BMI) || id:ebi-a-GCST007516	DR	16988	rs7177055	0.03296	0.0074	8E-06
Type 2 diabetes (adjusted for BMI) || id:ebi-a-GCST007516	DR	16988	rs7202877	0.03572	0.0078	5E-06
Type 2 diabetes (adjusted for BMI) || id:ebi-a-GCST007516	DR	16988	rs730497	0.03577	0.0077	4E-06

Type 2 diabetes (adjusted for BMI) || id:ebi-a-GCST007516	DR	16988	rs731839	0.03658	0.0075	1E-06
Type 2 diabetes (adjusted for BMI) || id:ebi-a-GCST007516	DR	16988	rs738409	0.03556	0.0078	6E-06
Type 2 diabetes (adjusted for BMI) || id:ebi-a-GCST007516	DR	16988	rs7501939	0.03381	0.0078	2E-05
Type 2 diabetes (adjusted for BMI) || id:ebi-a-GCST007516	DR	16988	rs7756992	0.03575	0.008	9E-06
Type 2 diabetes (adjusted for BMI) || id:ebi-a-GCST007516	DR	16988	rs7903146	0.03678	0.0091	5E-05
Type 2 diabetes (adjusted for BMI) || id:ebi-a-GCST007516	DR	16988	rs8042680	0.03587	0.0078	4E-06
Type 2 diabetes (adjusted for BMI) || id:ebi-a-GCST007516	DR	16988	rs8108269	0.03453	0.0079	1E-05
Type 2 diabetes (adjusted for BMI) || id:ebi-a-GCST007516	DR	16988	rs864745	0.03428	0.0079	2E-05
Type 2 diabetes (adjusted for BMI) || id:ebi-a-GCST007516	DR	16988	rs9379084	0.03496	0.0079	1E-05
Type 2 diabetes (adjusted for BMI) || id:ebi-a-GCST007516	DR	16988	rs9388489	0.03492	0.0079	9E-06
Type 2 diabetes (adjusted for BMI) || id:ebi-a-GCST007516	DR	16988	rs972283	0.0357	0.0078	5E-06
Type 2 diabetes (adjusted for BMI) || id:ebi-a-GCST007516	DR	16988	All	0.03503	0.0078	7E-06
Proinsulin						
**exposure**	**outcome**	**method**	**nsnp**	**b**	**se**	**pval**
Proinsulin levels || id:ebi-a-GCST001212	DR	Inverse variance weighted	8	0.05428	0.0167	0.001
Proinsulin levels || id:ebi-a-GCST001212	DR	MR Egger	8	0.06051	0.0413	0.193
Proinsulin levels || id:ebi-a-GCST001212	DR	Weighted median	8	0.04322	0.0227	0.056
Proinsulin levels || id:ebi-a-GCST001212	DR	Simple median	8	0.03791	0.0236	0.109
Proinsulin levels || id:ebi-a-GCST001212	DR	Weighted mode	8	0.04668	0.0279	0.138
**heterogeneity statistics results**						
**exposure**	**outcome**	**method**	**Q**	**Q_df**	**Q_pval**	
Proinsulin levels || id:ebi-a-GCST001212	DR	Inverse variance weighted	6.607572098	7	0.4708	
Proinsulin levels || id:ebi-a-GCST001212	DR	MR Egger	6.577220933	6	0.3617	
**Horizontal pleiotropy results**						

exposure	outcome	egger_intercept	se	pval		
Proinsulin levels || id:ebi-a-GCST001212	DR	-0.000551244	0.00331285	0.87331		
**Single SNP analysis results**						
**exposure**	**outcome**	**samplesize**	**SNP**	**b**	**se**	**p**
Proinsulin levels || id:ebi-a-GCST001212	DR	16988	rs10501320	0.09124	0.0343	0.008
Proinsulin levels || id:ebi-a-GCST001212	DR	16988	rs11558471	0.00448	0.0639	0.944
Proinsulin levels || id:ebi-a-GCST001212	DR	16988	rs11603334	0.03265	0.0368	0.375
Proinsulin levels || id:ebi-a-GCST001212	DR	16988	rs4502156	0.12483	0.0697	0.073
Proinsulin levels || id:ebi-a-GCST001212	DR	16988	rs4790333	0.01062	0.0832	0.898
Proinsulin levels || id:ebi-a-GCST001212	DR	16988	rs6101962	0.04318	0.0352	0.221
Proinsulin levels || id:ebi-a-GCST001212	DR	16988	rs6235	-0.0379	0.0643	0.556
Proinsulin levels || id:ebi-a-GCST001212	DR	16988	rs7903146	0.10699	0.0517	0.039
Proinsulin levels || id:ebi-a-GCST001212	DR	16988	All - Inverse variance weighted	0.05428	0.0167	0.001
Proinsulin levels || id:ebi-a-GCST001212	DR	16988	All - MR Egger	0.06051	0.0413	0.193
**Leave-one-out analysis results**						
**exposure**	**outcome**	**samplesize**	**SNP**	**b**	**se**	**p**
Proinsulin levels || id:ebi-a-GCST001212	DR	16988	rs10501320	0.04287	0.0191	0.024
Proinsulin levels || id:ebi-a-GCST001212	DR	16988	rs11558471	0.05791	0.0172	8E-04
Proinsulin levels || id:ebi-a-GCST001212	DR	16988	rs11603334	0.05986	0.0189	0.002
Proinsulin levels || id:ebi-a-GCST001212	DR	16988	rs4502156	0.05001	0.0171	0.004
Proinsulin levels || id:ebi-a-GCST001212	DR	16988	rs4790333	0.05611	0.0174	0.001
Proinsulin levels || id:ebi-a-GCST001212	DR	16988	rs6101962	0.05748	0.0196	0.003
Proinsulin levels || id:ebi-a-GCST001212	DR	16988	rs6235	0.06091	0.0172	4E-04
Proinsulin levels || id:ebi-a-GCST001212	DR	16988	rs7903146	0.04819	0.0176	0.006

Proinsulin levels || id:ebi-a-GCST001212	DR	16988	All	0.05428	0.0167	0.001
Eye or eyelid problem						
**exposure**	**outcome**	**method**	**nsnp**	**b**	**se**	**pval**
Eye or eyelid problem || id:ebi-a-GCST90038640	DR	Inverse variance weighted	5	-1.414	0.514	0.006
Eye or eyelid problem || id:ebi-a-GCST90038640	DR	MR Egger	5	-1.333	1.088	0.308
Eye or eyelid problem || id:ebi-a-GCST90038640	DR	Weighted median	5	-1.519	0.653	0.020
Eye or eyelid problem || id:ebi-a-GCST90038640	DR	Simple median	5	-1.829	0.691	0.008
Eye or eyelid problem || id:ebi-a-GCST90038640	DR	Weighted mode	5	-1.475	0.778	0.131
**heterogeneity statistics results**						
**exposure**	**outcome**	**method**	**Q**	**Q_df**	**Q_pval**	
Eye or eyelid problem || id:ebi-a-GCST90038640	DR	Inverse variance weighted	1.909621532	4	0.752	
Eye or eyelid problem || id:ebi-a-GCST90038640	DR	MR Egger	1.902575705	3	0.593	
**Horizontal pleiotropy results**						
**exposure**	**outcome**	**egger_intercept**	**se**	**pval**		
Eye or eyelid problem || id:ebi-a-GCST90038640	DR	-0.000312469	0.003722554	0.938		
**Single SNP analysis results**						
**exposure**	**outcome**	**samplesize**	**SNP**	**b**	**se**	**p**
Eye or eyelid problem || id:ebi-a-GCST90038640	DR	16988	rs10757270	-1.849	1.388	0.183
Eye or eyelid problem || id:ebi-a-GCST90038640	DR	16988	rs146683910	-1.098	0.815	0.178
Eye or eyelid problem || id:ebi-a-GCST90038640	DR	16988	rs2472493	-1.829	1.173	0.119
Eye or eyelid problem || id:ebi-a-GCST90038640	DR	16988	rs2814471	-2.457	1.324	0.063
Eye or eyelid problem || id:ebi-a-GCST90038640	DR	16988	rs4669869	-0.003	1.475	0.998
Eye or eyelid problem || id:ebi-a-GCST90038640	DR	16988	All - Inverse variance weighted	-1.414	0.514	0.006
Eye or eyelid problem || id:ebi-a-GCST90038640	DR	16988	All - MR Egger	-1.333	1.088	0.308

Leave-one-out analysis results						
**exposure**	**outcome**	**samplesize**	**SNP**	**b**	**se**	**p**
Eye or eyelid problem || id:ebi-a-GCST90038640	DR	16988	rs10757270	-1.344	0.554	0.015
Eye or eyelid problem || id:ebi-a-GCST90038640	DR	16988	rs146683910	-1.622	0.663	0.014
Eye or eyelid problem || id:ebi-a-GCST90038640	DR	16988	rs2472493	-1.315	0.572	0.022
Eye or eyelid problem || id:ebi-a-GCST90038640	DR	16988	rs2814471	-1.228	0.558	0.028
Eye or eyelid problem || id:ebi-a-GCST90038640	DR	16988	rs4669869	-1.609	0.549	0.003
Eye or eyelid problem || id:ebi-a-GCST90038640	DR	16988	All	-1.414	0.514	0.006
Eye problems/disorders: Glaucoma						
**exposure**	**outcome**	**method**	**nsnp**	**b**	**se**	**pval**
Eye problems/disorders: Glaucoma || id:ukb-b-17324	DR	Inverse variance weighted	17	-0.366	0.145	0.012
Eye problems/disorders: Glaucoma || id:ukb-b-17324	DR	MR Egger	17	-0.324	0.441	0.474
Eye problems/disorders: Glaucoma || id:ukb-b-17324	DR	Weighted median	17	-0.400	0.210	0.056
Eye problems/disorders: Glaucoma || id:ukb-b-17324	DR	Simple median	17	-0.333	0.205	0.105
Eye problems/disorders: Glaucoma || id:ukb-b-17324	DR	Weighted mode	17	-0.472	0.271	0.101
**heterogeneity statistics results**						
**exposure**	**outcome**	**method**	**Q**	**Q_df**	**Q_pval**	
Eye problems/disorders: Glaucoma || id:ukb-b-17324	DR	Inverse variance weighted	14.78817973	16	0.540	
Eye problems/disorders: Glaucoma || id:ukb-b-17324	DR	MR Egger	14.77821396	15	0.468	
**Horizontal pleiotropy results**						
**exposure**	**outcome**	**egger_intercept**	**se**	**pval**		
Eye problems/disorders: Glaucoma || id:ukb-b-17324	DR	-0.00027289	0.00273358	0.922		
**Single SNP analysis results**						
**exposure**	**outcome**	**samplesize**	**SNP**	**b**	**se**	**p**

Eye problems/disorders: Glaucoma || id:ukb-b-17324	DR	16988	rs12699251	-0.020	0.777	0.980
Eye problems/disorders: Glaucoma || id:ukb-b-17324	DR	16988	rs1556515	-0.588	0.478	0.219
Eye problems/disorders: Glaucoma || id:ukb-b-17324	DR	16988	rs2024211	-1.055	0.815	0.195
Eye problems/disorders: Glaucoma || id:ukb-b-17324	DR	16988	rs2073006	1.201	0.694	0.083
Eye problems/disorders: Glaucoma || id:ukb-b-17324	DR	16988	rs2472493	-0.852	0.546	0.119
Eye problems/disorders: Glaucoma || id:ukb-b-17324	DR	16988	rs2514884	0.580	0.699	0.407
Eye problems/disorders: Glaucoma || id:ukb-b-17324	DR	16988	rs2814471	-0.594	0.320	0.063
Eye problems/disorders: Glaucoma || id:ukb-b-17324	DR	16988	rs34935520	-0.261	0.631	0.679
Eye problems/disorders: Glaucoma || id:ukb-b-17324	DR	16988	rs55937650	-0.330	0.507	0.515
Eye problems/disorders: Glaucoma || id:ukb-b-17324	DR	16988	rs62188040	-1.021	0.796	0.200
Eye problems/disorders: Glaucoma || id:ukb-b-17324	DR	16988	rs62578126	0.095	0.620	0.878
Eye problems/disorders: Glaucoma || id:ukb-b-17324	DR	16988	rs76325372	-0.333	0.743	0.654
Eye problems/disorders: Glaucoma || id:ukb-b-17324	DR	16988	rs8064739	-1.391	0.824	0.092
Eye problems/disorders: Glaucoma || id:ukb-b-17324	DR	16988	rs8142788	0.568	0.759	0.454
Eye problems/disorders: Glaucoma || id:ukb-b-17324	DR	16988	rs838720	-0.347	0.754	0.646
Eye problems/disorders: Glaucoma || id:ukb-b-17324	DR	16988	rs9284802	-1.135	0.772	0.141
Eye problems/disorders: Glaucoma || id:ukb-b-17324	DR	16988	rs9913911	-0.189	0.532	0.722
Eye problems/disorders: Glaucoma || id:ukb-b-17324	DR	16988	All - Inverse variance weighted	-0.366	0.145	0.012
Eye problems/disorders: Glaucoma || id:ukb-b-17324	DR	16988	All - MR Egger	-0.324	0.441	0.474
**Leave-one-out analysis results**						
**exposure**	**outcome**	**samplesize**	**SNP**	**b**	**se**	**p**
Eye problems/disorders: Glaucoma || id:ukb-b-17324	DR	16988	rs12699251	-0.378	0.148	0.011
Eye problems/disorders: Glaucoma || id:ukb-b-17324	DR	16988	rs1556515	-0.343	0.153	0.024
Eye problems/disorders: Glaucoma || id:ukb-b-17324	DR	16988	rs2024211	-0.343	0.148	0.020

Eye problems/disorders: Glaucoma || id:ukb-b-17324	DR	16988	rs2073006	-0.438	0.149	0.003
Eye problems/disorders: Glaucoma || id:ukb-b-17324	DR	16988	rs2472493	-0.329	0.151	0.029
Eye problems/disorders: Glaucoma || id:ukb-b-17324	DR	16988	rs2514884	-0.409	0.149	0.006
Eye problems/disorders: Glaucoma || id:ukb-b-17324	DR	16988	rs2814471	-0.307	0.163	0.060
Eye problems/disorders: Glaucoma || id:ukb-b-17324	DR	16988	rs34935520	-0.372	0.149	0.013
Eye problems/disorders: Glaucoma || id:ukb-b-17324	DR	16988	rs55937650	-0.369	0.152	0.015
Eye problems/disorders: Glaucoma || id:ukb-b-17324	DR	16988	rs62188040	-0.343	0.148	0.020
Eye problems/disorders: Glaucoma || id:ukb-b-17324	DR	16988	rs62578126	-0.393	0.149	0.009
Eye problems/disorders: Glaucoma || id:ukb-b-17324	DR	16988	rs76325372	-0.367	0.148	0.013
Eye problems/disorders: Glaucoma || id:ukb-b-17324	DR	16988	rs8064739	-0.333	0.148	0.024
Eye problems/disorders: Glaucoma || id:ukb-b-17324	DR	16988	rs8142788	-0.401	0.148	0.007
Eye problems/disorders: Glaucoma || id:ukb-b-17324	DR	16988	rs838720	-0.367	0.148	0.013
Eye problems/disorders: Glaucoma || id:ukb-b-17324	DR	16988	rs9284802	-0.338	0.148	0.023
Eye problems/disorders: Glaucoma || id:ukb-b-17324	DR	16988	rs9913911	-0.380	0.151	0.012
Eye problems/disorders: Glaucoma || id:ukb-b-17324	DR	16988	All	-0.366	0.145	0.012

Two-sample Mendelian randomization was performed in R using the TwoSampleMR package. Causal effects were estimated using inverse-variance weighted (IVW; multiplicative random-effects), MR-Egger regression, weighted median, simple median, and weighted mode methods. For each estimator, statistical significance was evaluated using a two-sided z-test of the causal estimate (BETA/SE) against the null hypothesis BETA = 0, and 95% confidence intervals were reported; standard errors for median/mode estimators were obtained by bootstrapping (default settings). Between-instrument heterogeneity was assessed using Cochran's Q statistic. Directional horizontal pleiotropy was assessed using the MR-Egger intercept test (two-sided test of intercept = 0). Sensitivity analyses included single-SNP Wald ratio estimates and leave-one-out analysis (IVW re-estimated after removing one SNP at a time). *P* values are nominal and were not adjusted for multiple comparisons.

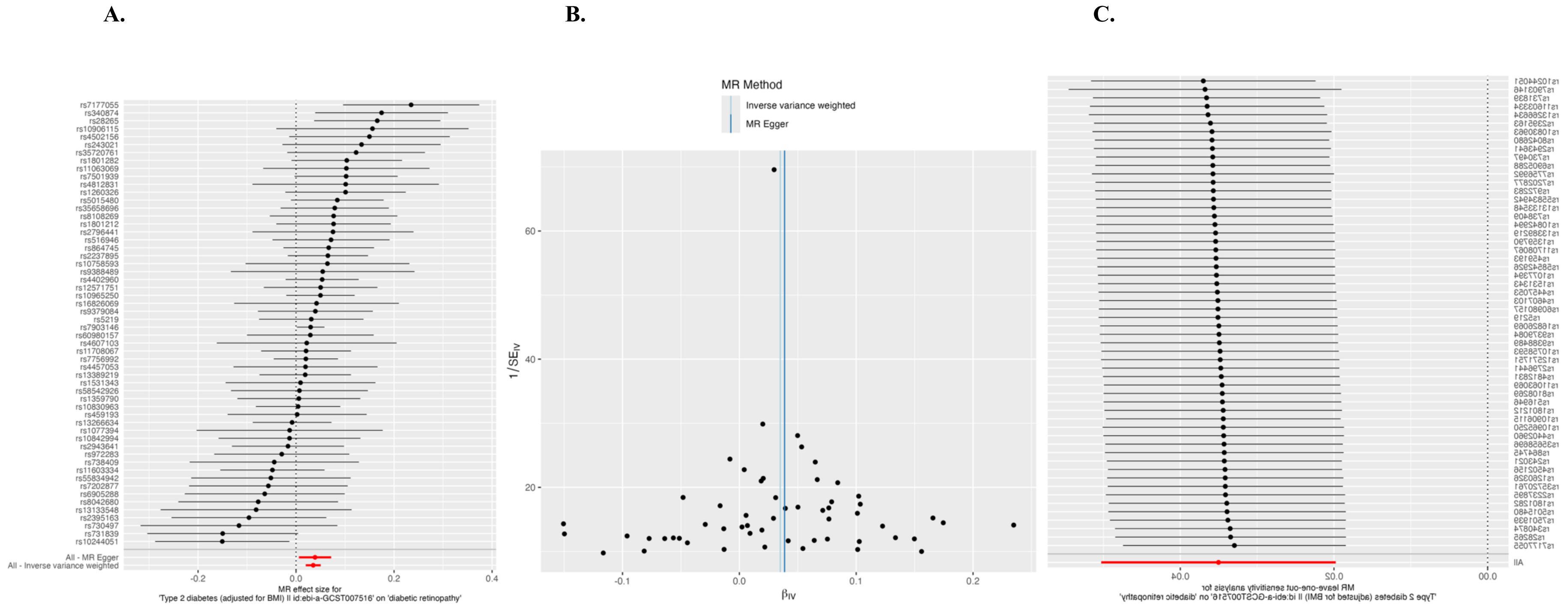
A.
B.
C.
All - MR Egger
All - Inverse variance weighted
MR effect size for
'Type 2 diabetes (adjusted for BMI) || id:ebi-a-GCST007516' on 'diabetic retinopathy'
MR Method
Inverse variance weighted
MR Egger
1/SE_IV
β_IV
MR leave-one-out sensitivity analysis for
'Type 2 diabetes (adjusted for BMI) || id:ebi-a-GCST007516' on 'diabetic retinopathy'

D. **E.** **F.**

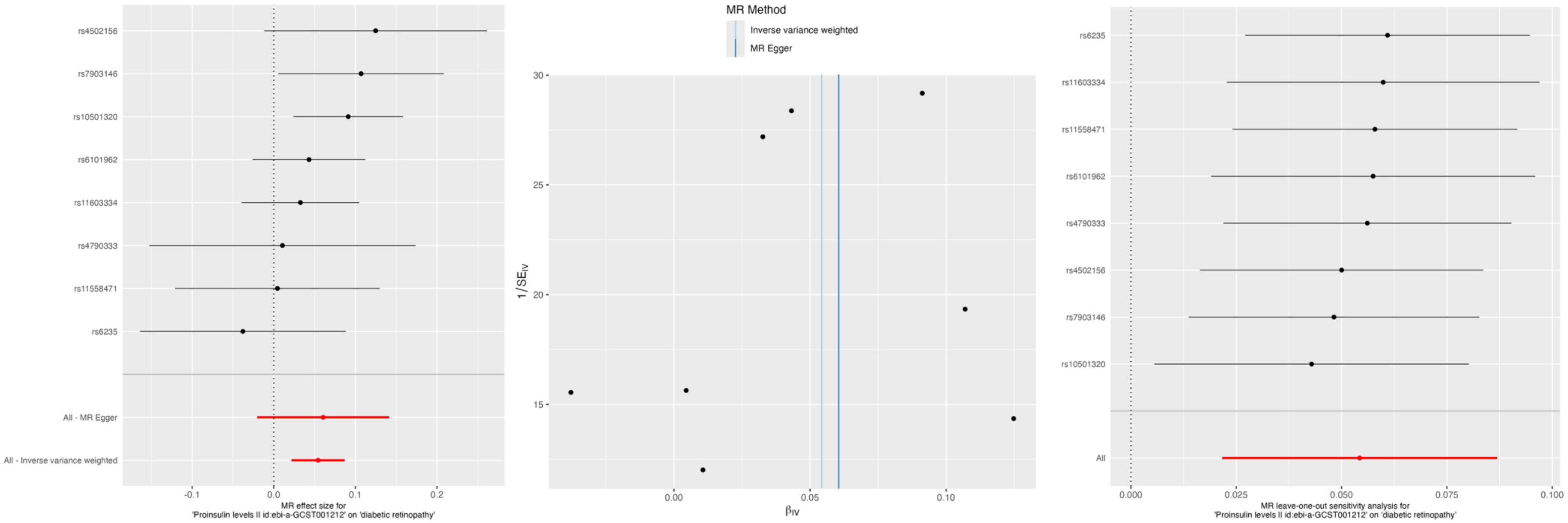

rs4502156
rs7903146
rs10501320
rs6101962
rs11603334
rs4790333
rs11558471
rs6235
All - MR Egger
All - Inverse variance weighted
-0.1
0.0
0.1
0.2
MR effect size for
'Proinsulin levels II id:ebi-a-GCST001212' on 'diabetic retinopathy'
MR Method
Inverse variance weighted
MR Egger
30
25
20
15
1/SE$_{IV}$
0.00
0.05
0.10
β$_{IV}$
rs6235
rs11603334
rs11558471
rs6101962
rs4790333
rs4502156
rs7903146
rs10501320
All
0.000
0.025
0.050
0.075
0.100
MR leave-one-out sensitivity analysis for
'Proinsulin levels II id:ebi-a-GCST001212' on 'diabetic retinopathy'

G.

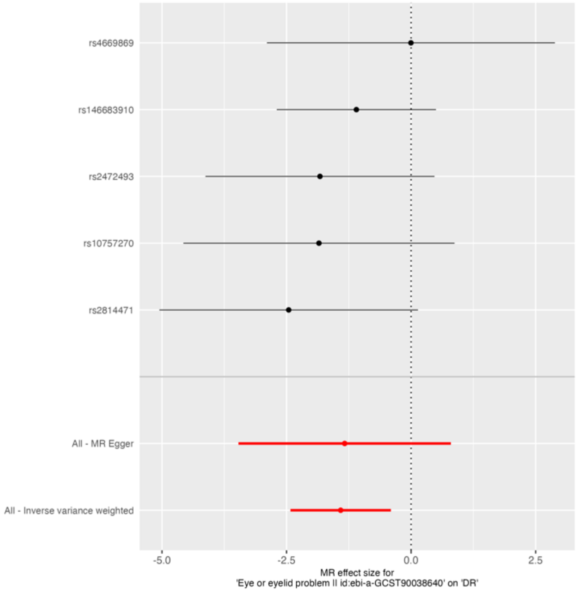
rs4669869
rs146683910
rs2472493
rs10757270
rs2814471
All - MR Egger
All - Inverse variance weighted
-5.0
-2.5
0.0
2.5
MR effect size for
'Eye or eyelid problem II id:ebi-a-GCST90038640' on 'DR'

H.

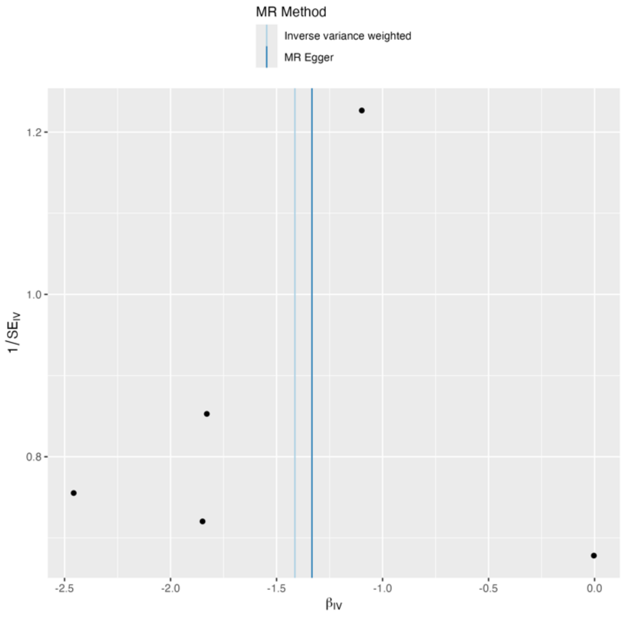
MR Method
Inverse variance weighted
MR Egger
1.2
1.0
0.8
$1/SE_{IV}$
-2.5
-2.0
-1.5
-1.0
-0.5
0.0
β_{IV}

I.

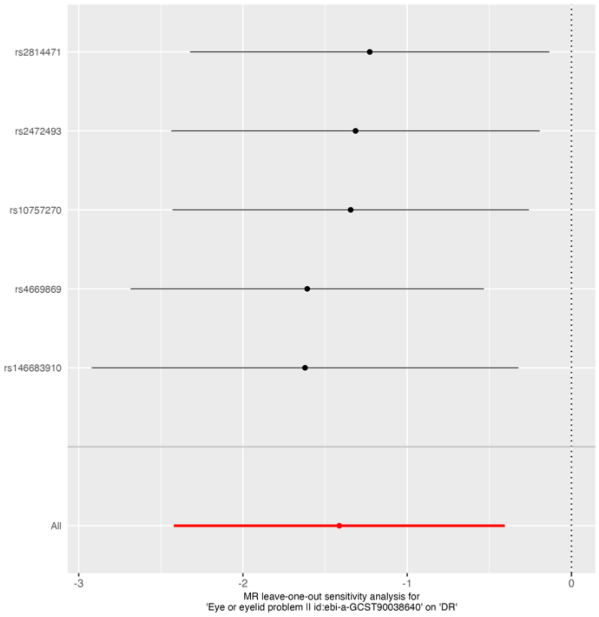
rs2814471
rs2472493
rs10757270
rs4669869
rs146683910
All
-3
-2
-1
0
MR leave-one-out sensitivity analysis for
'Eye or eyelid problem II id:ebi-a-GCST90038640' on 'DR'

J. **K.** **L.**

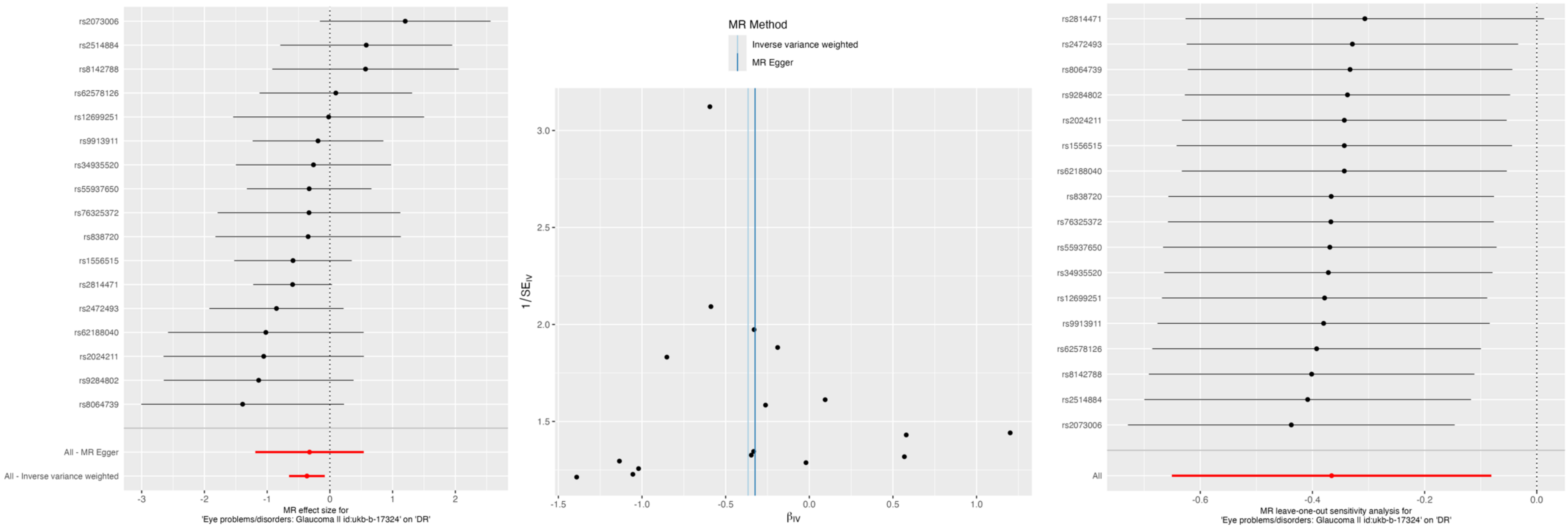

Supplementary Figure 5. Sensitivity analyses of the plot of Mendelian Randomization(MR) results between T2D adjusted for BMI(A-C)/proinsulin(D-F)/eyelid problem (G-I)/glaucoma (J-L) and diabetic retinopathy(DR).

Panels A & D & G & J: Forest Plots depicting the estimated causal effects of specific traits on DR. Black points with horizontal lines represent single-SNP estimates and their 95% confidence intervals. Red points and lines illustrate the combined multi-SNP estimates derived from the inverse-variance weighted and MR-Egger methods. The dotted vertical line indicates the null effect (zero).

Panels B & E & H & K: Funnel Plots displaying single-SNP MR estimates, with effect sizes (β_IV) on the x-axis and precision ($1/SE\_IV$) on the y-axis. Vertical lines denote summary estimates from the inverse-variance weighted (light line) and MR-Egger (dark line) methods.

Panels C & F & I & L: Leave-One-Out Sensitivity Analyses for specific traits on DR. Black points with 95% confidence intervals show the causal effect estimates when each SNP is individually excluded. The red point and line represent the overall estimate using all SNPs. The dotted vertical line signifies the null effect, demonstrating the robustness of MR estimates with minimal variation upon SNP exclusion.

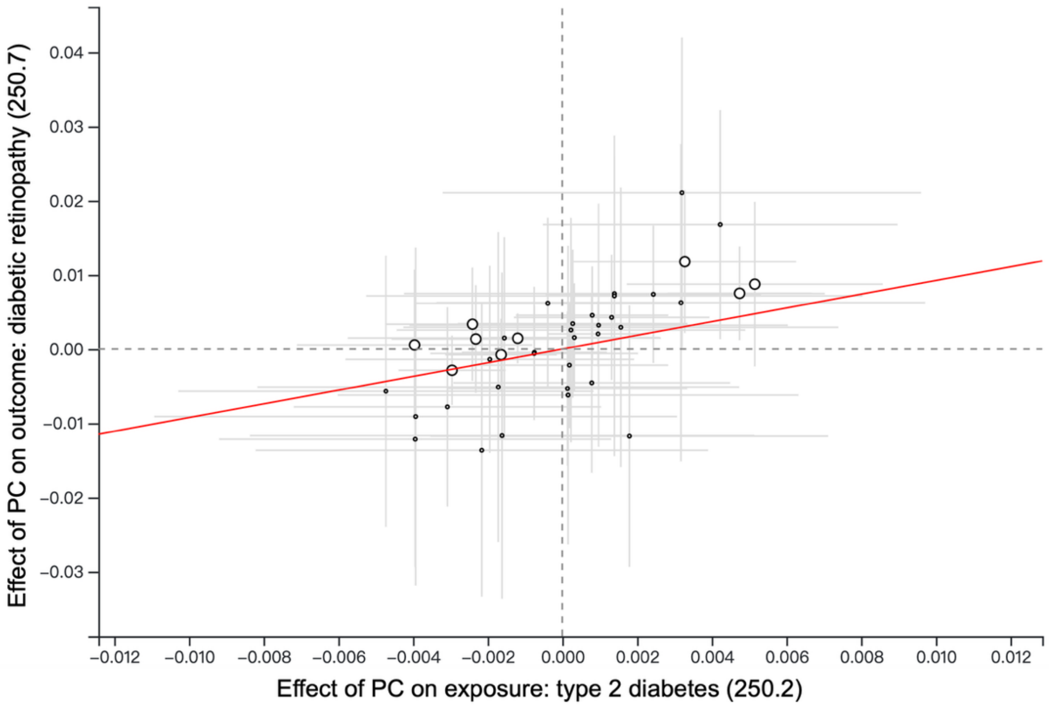

Supplementary Figure 6. Cis-MR analysis of ExPheWas-derived instruments at the *EYA2* locus

Variant-level associations of genetic instruments (cis-variants near *EYA2*) for a principal component reflecting type 2 diabetes liability (phecode 250.2) against diabetic retinopathy risk (phecode 250.7), both derived from ExPheWas. Horizontal and vertical bars denote SE of each variant's effect on exposure and outcome, respectively; point size is proportional to the inverse variance of the outcome estimate. The solid red line shows the inverse-variance weighted MR (IVW) causal estimate (OR = 2.52; 95% CI 1.22-5.21; P = 0.013). Vertical and horizontal dashed lines mark zero effect. The cis-association P-value for the *EYA2* variant on type 2 diabetes in ExPheWas is 3×10^{-7}.

Supplementary Table 13. Multivariate Mendelian Randomization results of BMI-adjusted T2D and proinsulin on DR.							
**id.exposure (IEU OpenGWAS ID)**	**exposure**	**id.outcome**	**outcome**	**nsnp**	**b**	**se**	**pval**
ebi-a-GCST001212	Proinsulin levels || id:ebi-a-GCST001212	diabetic retinopathy	diabetic retinopathy	3	0.0546	0.0259	0.0351
ebi-a-GCST007516	Type 2 diabetes (adjusted for BMI) || id:ebi-a-GCST007516	diabetic retinopathy	diabetic retinopathy	3	0.0175	0.0129	0.1729
For MVMR, we used the multivariable IVW method. Two-sided *p* values were derived from Wald z tests (BETA/SE). Given the limited number of prespecified MVMR tests, no multiple-testing correction was applied and $p < 0.05$ was considered significant.							

Supplementary Table 14: Mendelian Randomization Analysis of Four Eye Disorders on Diabetic Retinopathy						
Type 2 diabetes (adjusted for BMI) as exposure, proinsulin as outcome						
**exposure**	**outcome**	**method**	**nsnp**	**b**	**se**	**pval**
Type 2 diabetes (adjusted for BMI) || id:ebi-a-GCST007516	Proinsulin levels || id:ebi-a-GCST001212	Inverse variance weighted	47	0.060	0.027	0.025
Type 2 diabetes (adjusted for BMI) || id:ebi-a-GCST007516	Proinsulin levels || id:ebi-a-GCST001212	MR Egger	47	0.065	0.084	0.443
Type 2 diabetes (adjusted for BMI) || id:ebi-a-GCST007516	Proinsulin levels || id:ebi-a-GCST001212	Weighted median	47	0.056	0.033	0.089
Type 2 diabetes (adjusted for BMI) || id:ebi-a-GCST007516	Proinsulin levels || id:ebi-a-GCST001212	Simple median	47	0.056	0.034	0.099
Type 2 diabetes (adjusted for BMI) || id:ebi-a-GCST007516	Proinsulin levels || id:ebi-a-GCST001212	Weighted mode	47	0.079	0.060	0.193
**heterogeneity statistics results**						
**exposure**	**outcome**	**method**	**Q**	**Q_df**	**Q_pval**	
Type 2 diabetes (adjusted for BMI) || id:ebi-a-GCST007516	Proinsulin levels || id:ebi-a-GCST001212	Inverse variance weighted	89.126	47	0.0002	
Type 2 diabetes (adjusted for BMI) || id:ebi-a-GCST007516	Proinsulin levels || id:ebi-a-GCST001212	MR Egger	89.122	46	0.0001	
**Horizontal pleiotropy results**						
**exposure**	**outcome**	**egger_intercept**	**se**	**pval**		
Type 2 diabetes (adjusted for BMI) || id:ebi-a-GCST007516	Proinsulin levels || id:ebi-a-GCST001212	-0.0003	0.005	0.961		
**Single SNP analysis results**						
**exposure**	**outcome**	**samplesize**	**SNP**	**b**	**se**	**p**
Type 2 diabetes (adjusted for BMI) || id:ebi-a-GCST007516	Proinsulin levels || id:ebi-a-GCST001212	10701	rs10244051	0.251	0.148	0.091
Type 2 diabetes (adjusted for BMI) || id:ebi-a-GCST007516	Proinsulin levels || id:ebi-a-GCST001212	10701	rs10758593	0.280	0.181	0.121

Type 2 diabetes (adjusted for BMI) || id:ebi-a-GCST007516	Proinsulin levels || id:ebi-a-GCST001212	10701	rs1077394	-0.268	0.206	0.193
Type 2 diabetes (adjusted for BMI) || id:ebi-a-GCST007516	Proinsulin levels || id:ebi-a-GCST001212	10701	rs10830963	-0.229	0.106	0.031
Type 2 diabetes (adjusted for BMI) || id:ebi-a-GCST007516	Proinsulin levels || id:ebi-a-GCST001212	10701	rs10842994	0.144	0.155	0.351
Type 2 diabetes (adjusted for BMI) || id:ebi-a-GCST007516	Proinsulin levels || id:ebi-a-GCST001212	10701	rs10906115	-0.145	0.209	0.490
Type 2 diabetes (adjusted for BMI) || id:ebi-a-GCST007516	Proinsulin levels || id:ebi-a-GCST001212	10701	rs10965250	-0.017	0.073	0.817
Type 2 diabetes (adjusted for BMI) || id:ebi-a-GCST007516	Proinsulin levels || id:ebi-a-GCST001212	10701	rs11063069	-0.426	0.196	0.030
Type 2 diabetes (adjusted for BMI) || id:ebi-a-GCST007516	Proinsulin levels || id:ebi-a-GCST001212	10701	rs11708067	0.346	0.107	0.001
Type 2 diabetes (adjusted for BMI) || id:ebi-a-GCST007516	Proinsulin levels || id:ebi-a-GCST001212	10701	rs12571751	0.039	0.125	0.753
Type 2 diabetes (adjusted for BMI) || id:ebi-a-GCST007516	Proinsulin levels || id:ebi-a-GCST001212	10701	rs1260326	0.239	0.131	0.067
Type 2 diabetes (adjusted for BMI) || id:ebi-a-GCST007516	Proinsulin levels || id:ebi-a-GCST001212	10701	rs13133548	0.210	0.213	0.324
Type 2 diabetes (adjusted for BMI) || id:ebi-a-GCST007516	Proinsulin levels || id:ebi-a-GCST001212	10701	rs13389219	0.112	0.101	0.267
Type 2 diabetes (adjusted for BMI) || id:ebi-a-GCST007516	Proinsulin levels || id:ebi-a-GCST001212	10701	rs1359790	-0.222	0.133	0.096
Type 2 diabetes (adjusted for BMI) || id:ebi-a-GCST007516	Proinsulin levels || id:ebi-a-GCST001212	10701	rs1531343	-0.098	0.163	0.549
Type 2 diabetes (adjusted for BMI) || id:ebi-a-GCST007516	Proinsulin levels || id:ebi-a-GCST001212	10701	rs16826069	0.323	0.183	0.078
Type 2 diabetes (adjusted for BMI) || id:ebi-a-GCST007516	Proinsulin levels || id:ebi-a-GCST001212	10701	rs1801212	-0.286	0.124	0.021
Type 2 diabetes (adjusted for BMI) || id:ebi-a-GCST007516	Proinsulin levels || id:ebi-a-GCST001212	10701	rs1801282	-0.068	0.120	0.573

Type 2 diabetes (adjusted for BMI) || id:ebi-a-GCST007516	Proinsulin levels || id:ebi-a-GCST001212	10701	rs2395163	0.492	0.178	0.006
Type 2 diabetes (adjusted for BMI) || id:ebi-a-GCST007516	Proinsulin levels || id:ebi-a-GCST001212	10701	rs243021	0.398	0.177	0.024
Type 2 diabetes (adjusted for BMI) || id:ebi-a-GCST007516	Proinsulin levels || id:ebi-a-GCST001212	10701	rs2796441	0.275	0.208	0.185
Type 2 diabetes (adjusted for BMI) || id:ebi-a-GCST007516	Proinsulin levels || id:ebi-a-GCST001212	10701	rs28265	0.055	0.136	0.683
Type 2 diabetes (adjusted for BMI) || id:ebi-a-GCST007516	Proinsulin levels || id:ebi-a-GCST001212	10701	rs2943641	0.166	0.121	0.171
Type 2 diabetes (adjusted for BMI) || id:ebi-a-GCST007516	Proinsulin levels || id:ebi-a-GCST001212	10701	rs340874	-0.138	0.158	0.384
Type 2 diabetes (adjusted for BMI) || id:ebi-a-GCST007516	Proinsulin levels || id:ebi-a-GCST001212	10701	rs35720761	0.194	0.153	0.203
Type 2 diabetes (adjusted for BMI) || id:ebi-a-GCST007516	Proinsulin levels || id:ebi-a-GCST001212	10701	rs4457053	0.107	0.176	0.542
Type 2 diabetes (adjusted for BMI) || id:ebi-a-GCST007516	Proinsulin levels || id:ebi-a-GCST001212	10701	rs459193	0.026	0.151	0.863
Type 2 diabetes (adjusted for BMI) || id:ebi-a-GCST007516	Proinsulin levels || id:ebi-a-GCST001212	10701	rs4607103	-0.034	0.195	0.863
Type 2 diabetes (adjusted for BMI) || id:ebi-a-GCST007516	Proinsulin levels || id:ebi-a-GCST001212	10701	rs4812831	-0.221	0.221	0.317
Type 2 diabetes (adjusted for BMI) || id:ebi-a-GCST007516	Proinsulin levels || id:ebi-a-GCST001212	10701	rs5015480	0.006	0.100	0.955
Type 2 diabetes (adjusted for BMI) || id:ebi-a-GCST007516	Proinsulin levels || id:ebi-a-GCST001212	10701	rs516946	-0.153	0.124	0.217
Type 2 diabetes (adjusted for BMI) || id:ebi-a-GCST007516	Proinsulin levels || id:ebi-a-GCST001212	10701	rs5219	-0.222	0.117	0.059
Type 2 diabetes (adjusted for BMI) || id:ebi-a-GCST007516	Proinsulin levels || id:ebi-a-GCST001212	10701	rs55834942	-0.185	0.177	0.297
Type 2 diabetes (adjusted for BMI) || id:ebi-a-GCST007516	Proinsulin levels || id:ebi-a-GCST001212	10701	rs58542926	0.156	0.156	0.317

Type 2 diabetes (adjusted for BMI) || id:ebi-a-GCST007516	Proinsulin levels || id:ebi-a-GCST001212	10701	rs6813195	0.080	0.169	0.635
Type 2 diabetes (adjusted for BMI) || id:ebi-a-GCST007516	Proinsulin levels || id:ebi-a-GCST001212	10701	rs6905288	-0.022	0.182	0.902
Type 2 diabetes (adjusted for BMI) || id:ebi-a-GCST007516	Proinsulin levels || id:ebi-a-GCST001212	10701	rs7177055	0.184	0.146	0.208
Type 2 diabetes (adjusted for BMI) || id:ebi-a-GCST007516	Proinsulin levels || id:ebi-a-GCST001212	10701	rs7202877	0.344	0.165	0.037
Type 2 diabetes (adjusted for BMI) || id:ebi-a-GCST007516	Proinsulin levels || id:ebi-a-GCST001212	10701	rs730497	-0.291	0.228	0.202
Type 2 diabetes (adjusted for BMI) || id:ebi-a-GCST007516	Proinsulin levels || id:ebi-a-GCST001212	10701	rs731839	0.219	0.172	0.202
Type 2 diabetes (adjusted for BMI) || id:ebi-a-GCST007516	Proinsulin levels || id:ebi-a-GCST001212	10701	rs738409	-0.089	0.195	0.646
Type 2 diabetes (adjusted for BMI) || id:ebi-a-GCST007516	Proinsulin levels || id:ebi-a-GCST001212	10701	rs7756992	0.149	0.074	0.043
Type 2 diabetes (adjusted for BMI) || id:ebi-a-GCST007516	Proinsulin levels || id:ebi-a-GCST001212	10701	rs8042680	0.075	0.173	0.665
Type 2 diabetes (adjusted for BMI) || id:ebi-a-GCST007516	Proinsulin levels || id:ebi-a-GCST001212	10701	rs8108269	0.258	0.162	0.112
Type 2 diabetes (adjusted for BMI) || id:ebi-a-GCST007516	Proinsulin levels || id:ebi-a-GCST001212	10701	rs864745	0.056	0.098	0.572
Type 2 diabetes (adjusted for BMI) || id:ebi-a-GCST007516	Proinsulin levels || id:ebi-a-GCST001212	10701	rs9379084	0.275	0.116	0.018
Type 2 diabetes (adjusted for BMI) || id:ebi-a-GCST007516	Proinsulin levels || id:ebi-a-GCST001212	10701	rs9388489	-0.078	0.206	0.704
Type 2 diabetes (adjusted for BMI) || id:ebi-a-GCST007516	Proinsulin levels || id:ebi-a-GCST001212	10701	rs972283	0.042	0.150	0.778
Type 2 diabetes (adjusted for BMI) || id:ebi-a-GCST007516	Proinsulin levels || id:ebi-a-GCST001212	10701	All - Inverse variance weighted	0.051	0.027	0.061
Type 2 diabetes (adjusted for BMI) || id:ebi-a-GCST007516	Proinsulin levels || id:ebi-a-GCST001212	10701	All - MR Egger	0.055	0.087	0.527

Leave-one-out analysis results						
**exposure**	**outcome**	**samplesize**	**SNP**	**b**	**se**	**p**
Type 2 diabetes (adjusted for BMI) || id:ebi-a-GCST007516	Proinsulin levels || id:ebi-a-GCST001212	10701	rs10244051	0.048	0.028	0.085
Type 2 diabetes (adjusted for BMI) || id:ebi-a-GCST007516	Proinsulin levels || id:ebi-a-GCST001212	10701	rs10758593	0.049	0.028	0.080
Type 2 diabetes (adjusted for BMI) || id:ebi-a-GCST007516	Proinsulin levels || id:ebi-a-GCST001212	10701	rs1077394	0.054	0.028	0.048
Type 2 diabetes (adjusted for BMI) || id:ebi-a-GCST007516	Proinsulin levels || id:ebi-a-GCST001212	10701	rs10830963	0.062	0.027	0.023
Type 2 diabetes (adjusted for BMI) || id:ebi-a-GCST007516	Proinsulin levels || id:ebi-a-GCST001212	10701	rs10842994	0.050	0.028	0.075
Type 2 diabetes (adjusted for BMI) || id:ebi-a-GCST007516	Proinsulin levels || id:ebi-a-GCST001212	10701	rs10906115	0.053	0.028	0.055
Type 2 diabetes (adjusted for BMI) || id:ebi-a-GCST007516	Proinsulin levels || id:ebi-a-GCST001212	10701	rs10965250	0.057	0.029	0.048
Type 2 diabetes (adjusted for BMI) || id:ebi-a-GCST007516	Proinsulin levels || id:ebi-a-GCST001212	10701	rs11063069	0.056	0.027	0.037
Type 2 diabetes (adjusted for BMI) || id:ebi-a-GCST007516	Proinsulin levels || id:ebi-a-GCST001212	10701	rs11708067	0.041	0.027	0.131
Type 2 diabetes (adjusted for BMI) || id:ebi-a-GCST007516	Proinsulin levels || id:ebi-a-GCST001212	10701	rs12571751	0.052	0.028	0.066
Type 2 diabetes (adjusted for BMI) || id:ebi-a-GCST007516	Proinsulin levels || id:ebi-a-GCST001212	10701	rs1260326	0.047	0.028	0.091
Type 2 diabetes (adjusted for BMI) || id:ebi-a-GCST007516	Proinsulin levels || id:ebi-a-GCST001212	10701	rs13133548	0.050	0.028	0.072
Type 2 diabetes (adjusted for BMI) || id:ebi-a-GCST007516	Proinsulin levels || id:ebi-a-GCST001212	10701	rs13389219	0.049	0.028	0.084
Type 2 diabetes (adjusted for BMI) || id:ebi-a-GCST007516	Proinsulin levels || id:ebi-a-GCST001212	10701	rs1359790	0.058	0.027	0.035
Type 2 diabetes (adjusted for BMI) || id:ebi-a-GCST007516	Proinsulin levels || id:ebi-a-GCST001212	10701	rs1531343	0.054	0.028	0.054

Type 2 diabetes (adjusted for BMI) || id:ebi-a-GCST007516	Proinsulin levels || id:ebi-a-GCST001212	10701	rs16826069	0.048	0.028	0.081
Type 2 diabetes (adjusted for BMI) || id:ebi-a-GCST007516	Proinsulin levels || id:ebi-a-GCST001212	10701	rs1801212	0.060	0.027	0.025
Type 2 diabetes (adjusted for BMI) || id:ebi-a-GCST007516	Proinsulin levels || id:ebi-a-GCST001212	10701	rs1801282	0.055	0.028	0.051
Type 2 diabetes (adjusted for BMI) || id:ebi-a-GCST007516	Proinsulin levels || id:ebi-a-GCST001212	10701	rs2395163	0.046	0.027	0.089
Type 2 diabetes (adjusted for BMI) || id:ebi-a-GCST007516	Proinsulin levels || id:ebi-a-GCST001212	10701	rs243021	0.047	0.027	0.086
Type 2 diabetes (adjusted for BMI) || id:ebi-a-GCST007516	Proinsulin levels || id:ebi-a-GCST001212	10701	rs2796441	0.049	0.028	0.075
Type 2 diabetes (adjusted for BMI) || id:ebi-a-GCST007516	Proinsulin levels || id:ebi-a-GCST001212	10701	rs28265	0.051	0.028	0.068
Type 2 diabetes (adjusted for BMI) || id:ebi-a-GCST007516	Proinsulin levels || id:ebi-a-GCST001212	10701	rs2943641	0.048	0.028	0.085
Type 2 diabetes (adjusted for BMI) || id:ebi-a-GCST007516	Proinsulin levels || id:ebi-a-GCST001212	10701	rs340874	0.054	0.028	0.050
Type 2 diabetes (adjusted for BMI) || id:ebi-a-GCST007516	Proinsulin levels || id:ebi-a-GCST001212	10701	rs35720761	0.049	0.028	0.079
Type 2 diabetes (adjusted for BMI) || id:ebi-a-GCST007516	Proinsulin levels || id:ebi-a-GCST001212	10701	rs4457053	0.051	0.028	0.070
Type 2 diabetes (adjusted for BMI) || id:ebi-a-GCST007516	Proinsulin levels || id:ebi-a-GCST001212	10701	rs459193	0.052	0.028	0.064
Type 2 diabetes (adjusted for BMI) || id:ebi-a-GCST007516	Proinsulin levels || id:ebi-a-GCST001212	10701	rs4607103	0.052	0.028	0.061
Type 2 diabetes (adjusted for BMI) || id:ebi-a-GCST007516	Proinsulin levels || id:ebi-a-GCST001212	10701	rs4812831	0.054	0.028	0.052
Type 2 diabetes (adjusted for BMI) || id:ebi-a-GCST007516	Proinsulin levels || id:ebi-a-GCST001212	10701	rs5015480	0.053	0.028	0.060
Type 2 diabetes (adjusted for BMI) || id:ebi-a-GCST007516	Proinsulin levels || id:ebi-a-GCST001212	10701	rs516946	0.057	0.028	0.040

Type 2 diabetes (adjusted for BMI) || id:ebi-a-GCST007516	Proinsulin levels || id:ebi-a-GCST001212	10701	rs5219	0.060	0.027	0.029
Type 2 diabetes (adjusted for BMI) || id:ebi-a-GCST007516	Proinsulin levels || id:ebi-a-GCST001212	10701	rs55834942	0.054	0.028	0.049
Type 2 diabetes (adjusted for BMI) || id:ebi-a-GCST007516	Proinsulin levels || id:ebi-a-GCST001212	10701	rs58542926	0.050	0.028	0.076
Type 2 diabetes (adjusted for BMI) || id:ebi-a-GCST007516	Proinsulin levels || id:ebi-a-GCST001212	10701	rs6813195	0.051	0.028	0.068
Type 2 diabetes (adjusted for BMI) || id:ebi-a-GCST007516	Proinsulin levels || id:ebi-a-GCST001212	10701	rs6905288	0.052	0.028	0.061
Type 2 diabetes (adjusted for BMI) || id:ebi-a-GCST007516	Proinsulin levels || id:ebi-a-GCST001212	10701	rs7177055	0.049	0.028	0.080
Type 2 diabetes (adjusted for BMI) || id:ebi-a-GCST007516	Proinsulin levels || id:ebi-a-GCST001212	10701	rs7202877	0.047	0.027	0.087
Type 2 diabetes (adjusted for BMI) || id:ebi-a-GCST007516	Proinsulin levels || id:ebi-a-GCST001212	10701	rs730497	0.054	0.028	0.050
Type 2 diabetes (adjusted for BMI) || id:ebi-a-GCST007516	Proinsulin levels || id:ebi-a-GCST001212	10701	rs731839	0.049	0.028	0.078
Type 2 diabetes (adjusted for BMI) || id:ebi-a-GCST007516	Proinsulin levels || id:ebi-a-GCST001212	10701	rs738409	0.053	0.028	0.058
Type 2 diabetes (adjusted for BMI) || id:ebi-a-GCST007516	Proinsulin levels || id:ebi-a-GCST001212	10701	rs7756992	0.044	0.029	0.126
Type 2 diabetes (adjusted for BMI) || id:ebi-a-GCST007516	Proinsulin levels || id:ebi-a-GCST001212	10701	rs8042680	0.051	0.028	0.068
Type 2 diabetes (adjusted for BMI) || id:ebi-a-GCST007516	Proinsulin levels || id:ebi-a-GCST001212	10701	rs8108269	0.048	0.028	0.082
Type 2 diabetes (adjusted for BMI) || id:ebi-a-GCST007516	Proinsulin levels || id:ebi-a-GCST001212	10701	rs864745	0.051	0.028	0.071
Type 2 diabetes (adjusted for BMI) || id:ebi-a-GCST007516	Proinsulin levels || id:ebi-a-GCST001212	10701	rs9379084	0.045	0.028	0.106
Type 2 diabetes (adjusted for BMI) || id:ebi-a-GCST007516	Proinsulin levels || id:ebi-a-GCST001212	10701	rs9388489	0.053	0.028	0.059

Type 2 diabetes (adjusted for BMI) || id:ebi-a-GCST007516	Proinsulin levels || id:ebi-a-GCST001212	10701	rs972283	0.052	0.028	0.066
Type 2 diabetes (adjusted for BMI) || id:ebi-a-GCST007516	Proinsulin levels || id:ebi-a-GCST001212	10701	All	0.051	0.027	0.061
proinsulin as exposure, Type 2 diabetes (adjusted for BMI) as outcome						
**exposure**	**outcome**	**method**	**nsnp**	**b**	**se**	**pval**
Proinsulin levels || id:ebi-a-GCST001212	Type 2 diabetes (adjusted for BMI) || id:ebi-a-GCST007516	Inverse variance weighted	7	0.505	0.558	0.366
Proinsulin levels || id:ebi-a-GCST001212	Type 2 diabetes (adjusted for BMI) || id:ebi-a-GCST007516	MR Egger	7	-0.908	1.425	0.552
Proinsulin levels || id:ebi-a-GCST001212	Type 2 diabetes (adjusted for BMI) || id:ebi-a-GCST007516	Weighted median	7	-0.069	0.067	0.305
Proinsulin levels || id:ebi-a-GCST001212	Type 2 diabetes (adjusted for BMI) || id:ebi-a-GCST007516	Simple median	7	0.353	0.138	0.011
Proinsulin levels || id:ebi-a-GCST001212	Type 2 diabetes (adjusted for BMI) || id:ebi-a-GCST007516	Weighted mode	7	-0.113	0.062	0.119
**heterogeneity statistics results**						
**exposure**	**outcome**	**method**	**Q**	**Q_df**	**Q_pval**	
Proinsulin levels || id:ebi-a-GCST001212	Type 2 diabetes (adjusted for BMI) || id:ebi-a-GCST007516	Inverse variance weighted	1345.826	6	1.299E-287	
Proinsulin levels || id:ebi-a-GCST001212	Type 2 diabetes (adjusted for BMI) || id:ebi-a-GCST007516	MR Egger	1093.146	5	4.077E-234	
**Horizontal pleiotropy results**						
**exposure**	**outcome**	**egger_intercept**	**se**	**pval**		
Proinsulin levels || id:ebi-a-GCST001212	Type 2 diabetes (adjusted for BMI) || id:ebi-a-GCST007516	0.112	0.104	0.331		
**Single SNP analysis results**						
**exposure**	**outcome**	**samplesize**	**SNP**	**b**	**se**	**p**
Proinsulin levels || id:ebi-a-GCST001212	Type 2 diabetes (adjusted for BMI) || id:ebi-a-GCST007516	298957	rs10501320	0.047	0.071	0.505

Proinsulin levels || id:ebi-a-GCST001212	Type 2 diabetes (adjusted for BMI) || id:ebi-a-GCST007516	298957	rs11558471	1.560	0.123	0.000
Proinsulin levels || id:ebi-a-GCST001212	Type 2 diabetes (adjusted for BMI) || id:ebi-a-GCST007516	298957	rs11603334	-0.678	0.069	0.000
Proinsulin levels || id:ebi-a-GCST001212	Type 2 diabetes (adjusted for BMI) || id:ebi-a-GCST007516	298957	rs4502156	0.833	0.135	0.000
Proinsulin levels || id:ebi-a-GCST001212	Type 2 diabetes (adjusted for BMI) || id:ebi-a-GCST007516	298957	rs4790333	-0.410	0.168	0.014
Proinsulin levels || id:ebi-a-GCST001212	Type 2 diabetes (adjusted for BMI) || id:ebi-a-GCST007516	298957	rs6235	0.353	0.128	0.006
Proinsulin levels || id:ebi-a-GCST001212	Type 2 diabetes (adjusted for BMI) || id:ebi-a-GCST007516	298957	rs7903146	3.597	0.103	0.000
Proinsulin levels || id:ebi-a-GCST001212	Type 2 diabetes (adjusted for BMI) || id:ebi-a-GCST007516	298957	All - Inverse variance weighted	0.505	0.558	0.366
Proinsulin levels || id:ebi-a-GCST001212	Type 2 diabetes (adjusted for BMI) || id:ebi-a-GCST007516	298957	All - MR Egger	-0.908	1.425	0.552
**Leave-one-out analysis results**						
**exposure**	**outcome**	**samplesize**	**SNP**	**b**	**se**	**p**
Proinsulin levels || id:ebi-a-GCST001212	Type 2 diabetes (adjusted for BMI) || id:ebi-a-GCST007516	298957	rs10501320	0.679	0.703	0.334
Proinsulin levels || id:ebi-a-GCST001212	Type 2 diabetes (adjusted for BMI) || id:ebi-a-GCST007516	298957	rs11558471	0.397	0.622	0.523
Proinsulin levels || id:ebi-a-GCST001212	Type 2 diabetes (adjusted for BMI) || id:ebi-a-GCST007516	298957	rs11603334	0.987	0.605	0.103
Proinsulin levels || id:ebi-a-GCST001212	Type 2 diabetes (adjusted for BMI) || id:ebi-a-GCST007516	298957	rs4502156	0.478	0.635	0.452
Proinsulin levels || id:ebi-a-GCST001212	Type 2 diabetes (adjusted for BMI) || id:ebi-a-GCST007516	298957	rs4790333	0.552	0.620	0.373
Proinsulin levels || id:ebi-a-GCST001212	Type 2 diabetes (adjusted for BMI) || id:ebi-a-GCST007516	298957	rs6235	0.519	0.639	0.417
Proinsulin levels || id:ebi-a-GCST001212	Type 2 diabetes (adjusted for BMI) || id:ebi-a-GCST007516	298957	rs7903146	0.038	0.314	0.903

Proinsulin levels || id:ebi-a-GCST001212	Type 2 diabetes (adjusted for BMI) || id:ebi-a-GCST007516	298957	All	0.505	0.558	0.366

Two-sample Mendelian randomization was performed in R using the TwoSampleMR package. Causal effects were estimated using inverse-variance weighted (IVW; multiplicative random-effects), MR-Egger regression, weighted median, simple median, and weighted mode methods. For each estimator, statistical significance was evaluated using a two-sided z-test of the causal estimate (BETA/SE) against the null hypothesis BETA = 0, and 95% confidence intervals were reported; standard errors for median/mode estimators were obtained by bootstrapping (default settings). Between-instrument heterogeneity was assessed using Cochran's Q statistic. Directional horizontal pleiotropy was assessed using the MR-Egger intercept test (two-sided test of intercept = 0). Sensitivity analyses included single-SNP Wald ratio estimates and leave-one-out analysis (IVW re-estimated after removing one SNP at a time). *P* values are nominal and were not adjusted for multiple comparisons.
For the MR of proinsulin (exposure) on BMI-adjusted T2D (outcome), the main MR estimates and corresponding sensitivity analyses results shown are from the analysis before MR-PRESSO outlier correction. After MR-PRESSO, only one instrument SNP remained, so MR methods and sensitivity analyses requiring multiple variants could not be calculated.

**Supplementary Table 15. Colocalization results for four traits and DR using summary statistics from GWAS or eQTL.**						
**Dataset1**	**Dataset2**	**PP.H0.abf**	**PP.H1.abf**	**PP.H2.abf**	**PP.H3.abf**	**PP.H4.abf**
T2D	DR	1.948E-07	1.429E-02	5.908E-06	4.330E-01	5.527E-01
proinsulin	DR	2.880E-02	5.200E-03	7.810E-01	1.410E-01	4.450E-02
thyroid_eQTL	DR	9.630E-21	1.430E-01	5.770E-20	8.550E-01	1.660E-03
spleen_eQTL	DR	/	/	/	/	/

PP.abf: Posterior Probability based on Approximate Bayes Factor

H0: Neither trait (dataset1, dataset2 = DR) shows an association signal.

H1: Only dataset1 shows a signal; dataset2 (DR) has no signal.

H2: Only dataset2 (DR) shows a signal; dataset1 has no signal.

H3: Both traits show signals, but they are driven by different causal variants.

H4: Both traits share the same causal variant (true colocalization).

*/ there is no signal of *EYA2* gene in spleen eQTL.

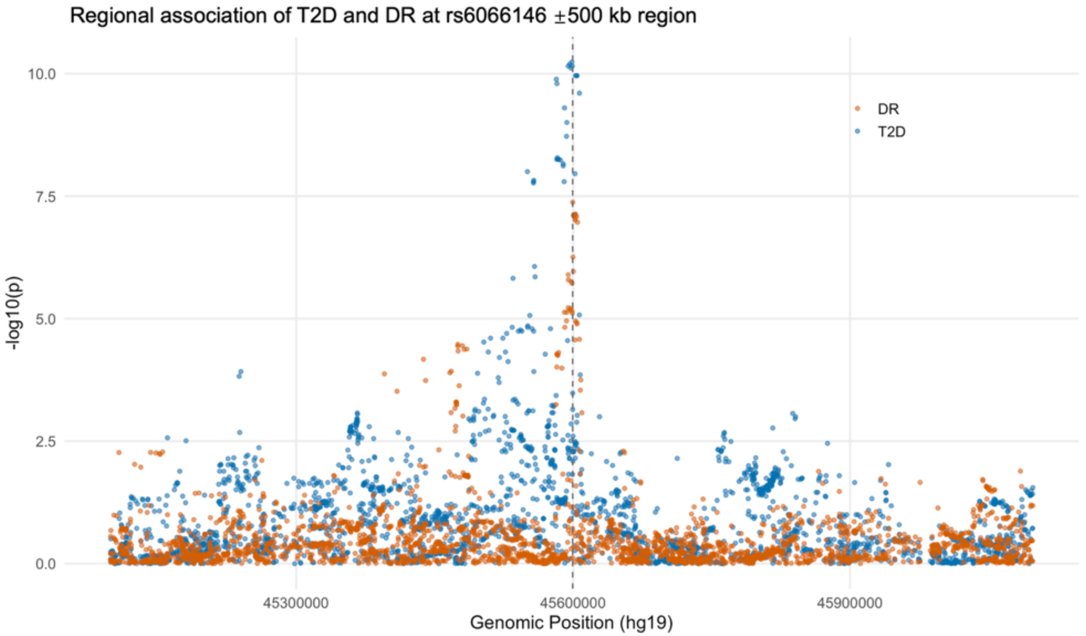

Supplementary Figure 7. Regional association plots of SNP across the selected region from the colocalization analysis

Shown are GWAS summary-statistic association signals for type 2 diabetes (T2D) and diabetic retinopathy (DR) within ±500 kb of the lead variant rs6066146. Panel shows the genomic position (hg19) on the x-axis and the association significance ($-\log_{10}p$) of individual SNPs on the y-axis. Blue and orange points represent different types of summary statistics, respectively. The vertical dashed line marks the lead SNP (rs6066146), and both traits show their strong local association signal in its vicinity.

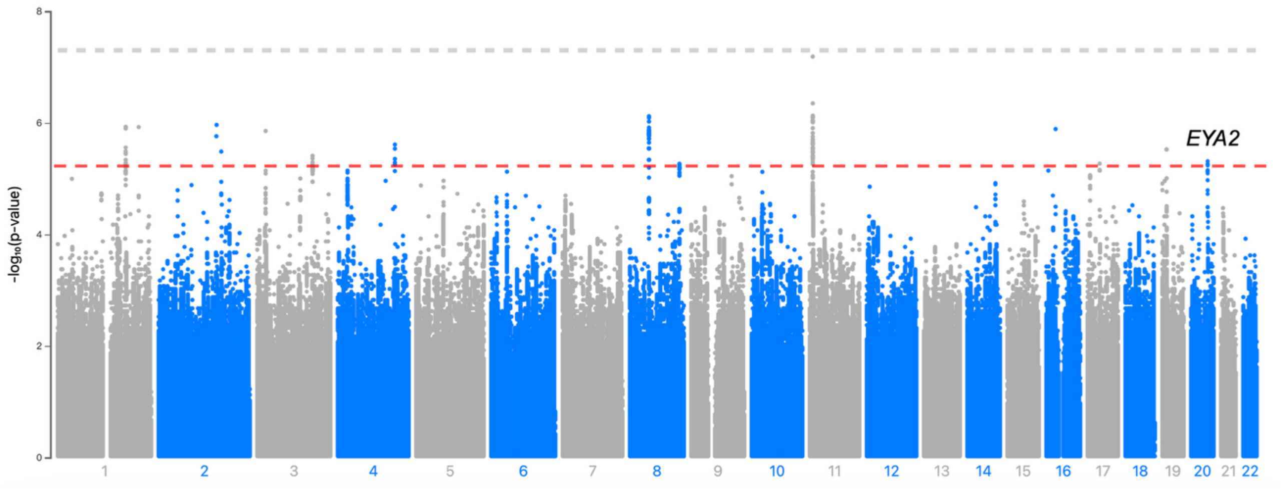

Supplementary Figure 8. Manhattan plot of GWAS for diabetic retinopathy excluding age-related macular degeneration cases

Each point corresponds to a single-nucleotide polymorphism (SNP) plotted by genomic position (hg19) on the x-axis and association significance ($-\log_{10}P$) on the y-axis. Chromosomes 1–22 are shown in alternating grey and blue. The grey dashed horizontal line marks the conventional genome-wide significance threshold ($P = 5 \times 10^{-8}$), and the red dashed line denotes the suggestive significance threshold ($P = 5 \times 10^{-6}$). SNP rs6066146 in *EYA2* was around suggestive significance threshold.

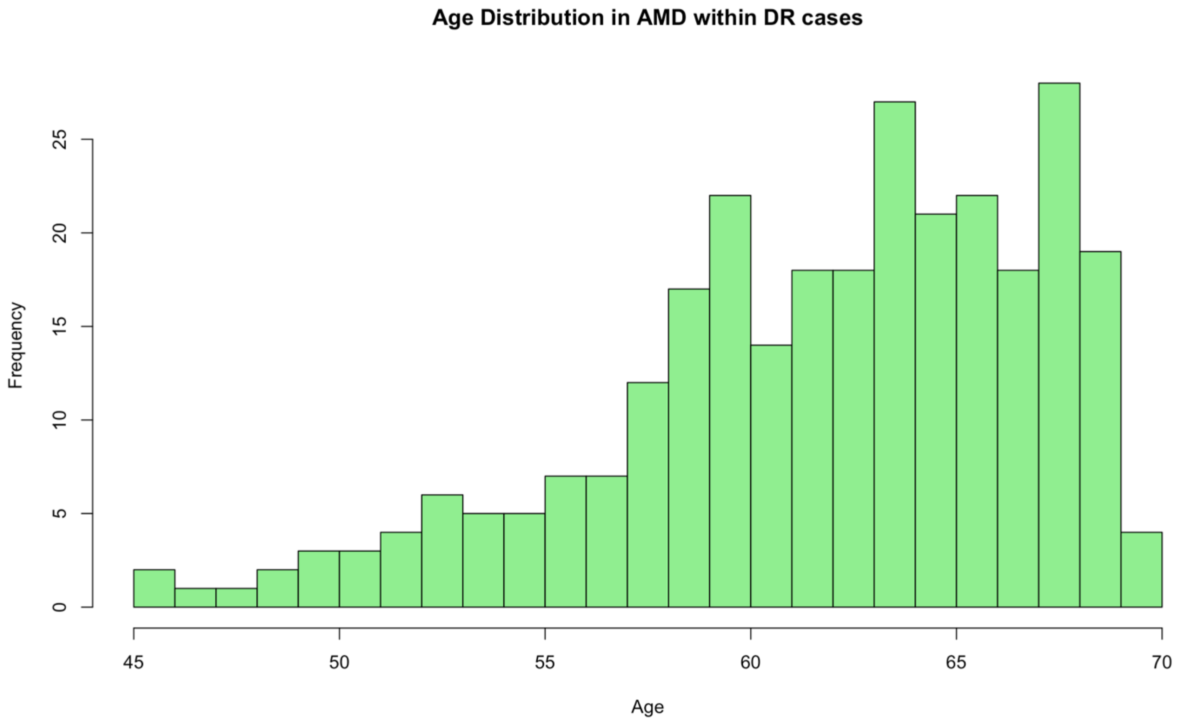

Supplementary Figure 9. Histogram of age distribution among diabetic retinopathy (DR) patients diagnosed with age-related macular degeneration (AMD).

The x-axis shows patient age (years) in 1-year bins from 45 to 70, and the y-axis indicates the number of DR cases presenting with AMD in each age bin. A total of N patients are included; frequencies rise steadily from the late 40s, peaking in the mid-60s, before declining slightly toward age 70.
