# Supplementary material for: A genome-wide association study identifies EYA2 as a contributing gene for diabetic retinopathy in type 2 diabetes
Source: Commun Med (Lond). 2026 Feb 25;6:181. doi: 10.1038/s43856-026-01465-1 (PMC13046729; doi:10.1038/s43856-026-01465-1)
Supplement: Supplementary file 3 — Description of Additional Supplementary Files [file 43856_2026_1465_MOESM3_ESM.docx]

**Description of Additional Supplementary Files**

Supplementary Data: This dataset is about the summary statistics of GWAS on diabetic retinopathy based on type 2 diabetes.
